# Immune-related events in individuals with solid tumors on immunotherapy associate with Th17 and Th2 signatures

**DOI:** 10.1172/JCI176567

**Published:** 2024-08-29

**Authors:** Chester J. Kao, Soren Charmsaz, Stephanie L. Alden, Madelena Brancati, Howard L. Li, Aanika Balaji, Kabeer Munjal, Kathryn Howe, Sarah Mitchell, James Leatherman, Ervin Griffin, Mari Nakazawa, Hua-Ling Tsai, Ludmila Danilova, Chris Thoburn, Jennifer Gizzi, Nicole E. Gross, Alexei Hernandez, Erin M. Coyne, Sarah M. Shin, Jayalaxmi Suresh Babu, George W. Apostol, Jennifer Durham, Brian J. Christmas, Maximilian F. Konig, Evan J. Lipson, Jarushka Naidoo, Laura C. Cappelli, Aliyah Pabani, Yasser Ged, Marina Baretti, Julie Brahmer, Jean Hoffman-Censits, Tanguy Y. Seiwert, Rachel Garonce-Hediger, Aditi Guha, Sanjay Bansal, Laura Tang, Elizabeth M. Jaffee, G. Scott Chandler, Rajat Mohindra, Won Jin Ho, Mark Yarchoan

**Affiliations:** 1 Sidney Kimmel Comprehensive Cancer Center, Johns Hopkins University, a member of the imCORE network, Baltimore, Maryland, USA.; 2 Johns Hopkins University School of Medicine, Baltimore, Maryland, USA.; 3 Convergence Institute and; 4 Bloomberg-Kimmel Institute for Cancer Immunotherapy, Johns Hopkins University, Baltimore, Maryland, USA.; 5 Division of Rheumatology, Department of Medicine, Johns Hopkins University School of Medicine, Baltimore, Maryland, USA.; 6 Beaumont Hospital, Dublin, Ireland.; 7 RCSI University of Health Sciences, Dublin, Ireland.; 8 F. Hoffmann-La Roche Ltd., a member of the imCORE network, Basel, Switzerland.; 9 Genentech Inc., a member of the imCORE network, South San Francisco, California, USA.

**Keywords:** Oncology, Cytokines, Immunotherapy, T cells

## Abstract

**BACKGROUND:**

Immune-related adverse events (irAEs) and their associated morbidity/mortality are a key concern for patients receiving immune checkpoint inhibitors (ICIs). Prospective evaluation of the drivers of irAEs in a diverse pan-tumor cohort is needed to identify patients at greatest risk and to develop rational treatment and interception strategies.

**METHODS:**

In an observational study, we prospectively collected blood samples and performed regular clinical evaluations for irAEs in patients receiving ICI therapy as standard of care for solid tumors. We performed in-parallel analysis of cytokines by Luminex immunoassay and circulating immune cells by cytometry by time-of-flight (CyTOF) at baseline and on treatment to investigate mechanisms of irAEs.

**RESULTS:**

We enrolled 111 patients, of whom 40.5% developed a symptomatic irAE (grade ≥ 2). Development of a grade ≥ 2 irAE was positively associated with the use of combination ICI and a history of an autoimmune disorder. Early changes in T helper 17 (Th17) (IL-6, IL-17f), type 2 (IL-5, IL-13, IL-25), and type 1 (TNF-α) cytokine signatures and congruent on-treatment expansions of Th17 and Th2 effector memory (Th2EM) T cell populations in peripheral blood were positively associated with the development of grade ≥2 irAEs. IL-6 levels were also associated with inferior cancer-specific survival and overall survival.

**CONCLUSIONS:**

In a diverse, prospective pan-tumor cohort, Th17 and Th2 skewing during early ICI treatment was associated with the development of clinically relevant irAEs but not antitumor responses, providing possible targets for monitoring and therapeutic interventions.

**FUNDING:**

Johns Hopkins Bloomberg-Kimmel Institute for Cancer Immunotherapy, the NCI SPORE in Gastrointestinal Cancers (P50 CA062924), NCI grant (R50CA243627 to LD), the NIH Center Core Grant (P30 CA006973), Swim Across America (to MY), NIAMS (K23AR075872 to LC), and imCORE-Genentech grant 137515 (to Johns Hopkins Medicine on behalf of MY).

## Abstract 2

In a diverse, prospective pan-tumor cohort, Th17 and Th2 skewing during early immunotherapy treatment was associated with the development of clinically relevant irAEs but not antitumor responses.

## Graphical abstract

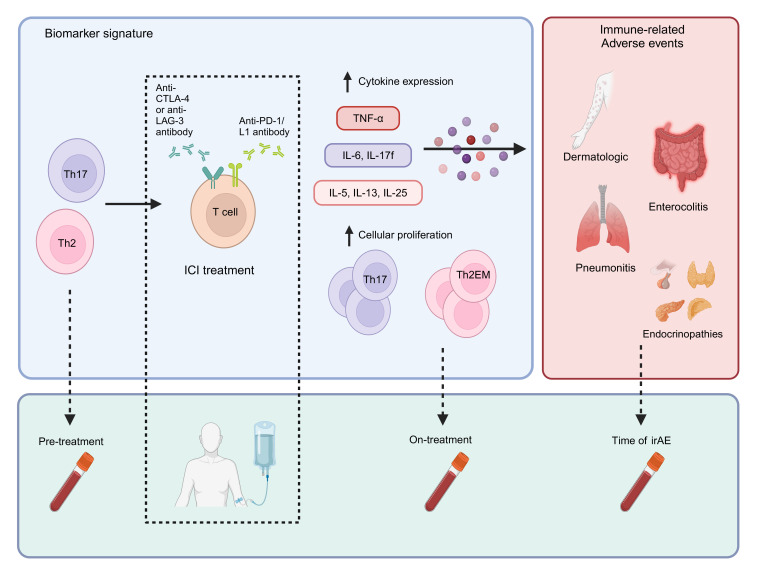

## Introduction

Immune checkpoint inhibitors (ICIs) can prolong life for patients with many types of cancers and have become firmly established as a major pillar of contemporary cancer care. However, these therapies can cause serious and sometimes fatal immune-related adverse events (irAEs) with a diverse range of presentations affecting multiple organs. ICIs reinvigorate tumor-directed T cell responses resulting in durable antitumor responses in a subset of patients (1). However, ICIs can also result in a loss of self tolerance resulting in irAEs (2). The 2 most widely used classes of ICI therapy are inhibitors of programmed cell death protein-1/ligand 1 (PD-1/L1) and cytotoxic T-lymphocyte-associated protein 4 (CTLA-4). Approximately, 60%–70% of patients receiving anti-PD-1 therapy develop an irAE, and, in combination with anti-CTLA-4, the rate of irAEs increases to approximately 90%, of which 40%–60% of events are severe or life-threatening (grade ≥3) (3–5). It is estimated that fatal irAEs associated with ICIs occur at a rate of 0.4%–1.2% in treated patients (6). As the use of ICI therapy increases, especially in patients with early stage malignancies, it is anticipated that irAEs will become an increasing cause of morbidity and mortality for patients with cancer. Therefore, irAEs are a key consideration when considering the benefits and risks of ICI therapies.

The relationship between responses to ICI and toxicity is complex; there is a modest but reproducible correlation between responses and toxicity. However, many patients achieving responses do not experience toxicity, and conversely, some patients develop toxicity without clinical benefit from therapy (7). This uncoupling of benefit and toxicity suggests that there are drivers of irAEs that are independent of antitumor responses. However, there are no validated biomarkers to identify patients at risk. These irAEs can affect any organ system, resulting in a unique challenge for clinical recognition and management (3). It is unlikely that a single mechanism can explain the full clinical spectrum of irAEs, and a diverse number of immunological mechanisms of irAEs have been proposed, including autoantibodies and amplification of preexisting B cell autoimmunity, drug-induced expansion of autoreactive T cell clones, dysregulated cytokine production, early B cell changes, and germline variants (8–15). IrAEs are usually identified clinically and are managed using paradigms adopted empirically from the treatment of spontaneous autoimmune disease (16). Although this is often an effective first-line strategy, some patients are refractory to current therapies, and indiscriminate blunting of T-cell responses with high-dose glucocorticoids may also impair antitumor responses (17–19). Improved understanding of the mechanisms that underpin these toxicities is needed to improve recognition, interception, and prevention of these adverse events without impeding antitumor immunity, ultimately improving outcomes for patients with cancer.

Given their relevance in autoimmune disorders, T helper cell (Th) subtypes and their associated cytokines are promising pathways for investigation into irAE-related autoimmunity. Biologics targeting specific cytokine pathways are widely used in the treatment of autoimmune diseases, including but not limited to Th2 cytokines (IL-4, IL-5, and IL-13), Th17 cytokines (IL-6, IL-17, and IL-23), and TNF-α. Biologics targeting Th2 response via type 2 cytokines such as IL-4 (dupilumab), IL-5 (benralizumab, mepolizumab, and reslizumab), and lL-13 (dupilumab and tralokinumab) are FDA approved for asthma and atopic dermatitis (20–22). Targeting Th17 cytokines such as IL-6, IL-17, and IL-23 is an effective strategy in rheumatoid arthritis, psoriasis, psoriatic arthritis, and inflammatory bowel disease (IBD) (23–26). Lastly, TNF-α blockade is effective in a number of autoimmune settings, including rheumatoid arthritis, psoriatic arthritis, ankylosing spondylitis, IBD, uveitis, and psoriasis (27). Active investigation into connecting these pathways to the development of irAEs from ICI is ongoing.

There are emerging data that certain cytokines and distinct T cell subtypes including IL-6 and Th17 are associated with specific irAEs (9–12, 28–30). These findings provide initial evidence that cytokines detected in peripheral blood may serve as the basis of rational prediction and interception strategies for irAEs. However, these prior studies are primarily derived from retrospective analyses or small interventional clinical trials in single cancer types, limiting generalizability. For example, prospective clinical trials have generally excluded patients with baseline autoimmune conditions, yet such patients experience cancer at increased rates and there exists an unmet need to provide them with treatment options (31). Additionally, the Black population has been significantly under represented, constituting less than 5% of patients in ICI clinical trials (32). Herein, we prospectively evaluated cytokines predictive of symptomatic irAEs (grade ≥2) at baseline and early on treatment as well as cytokine changes important at the time of irAE. We paired our cytokine analysis with in-depth characterization of circulating immune cell populations with cytometry by time-of-flight (CyTOF) to better understand the cellular basis of predictive cytokines in the development of symptomatic irAEs. Patients in this analysis represented a diverse pan-tumor cohort, which included those with and without preexisting autoimmune disease and were enriched with patients who identified as Black.

## Results

### Patient characteristics and irAE distribution.

**Figure 1:**
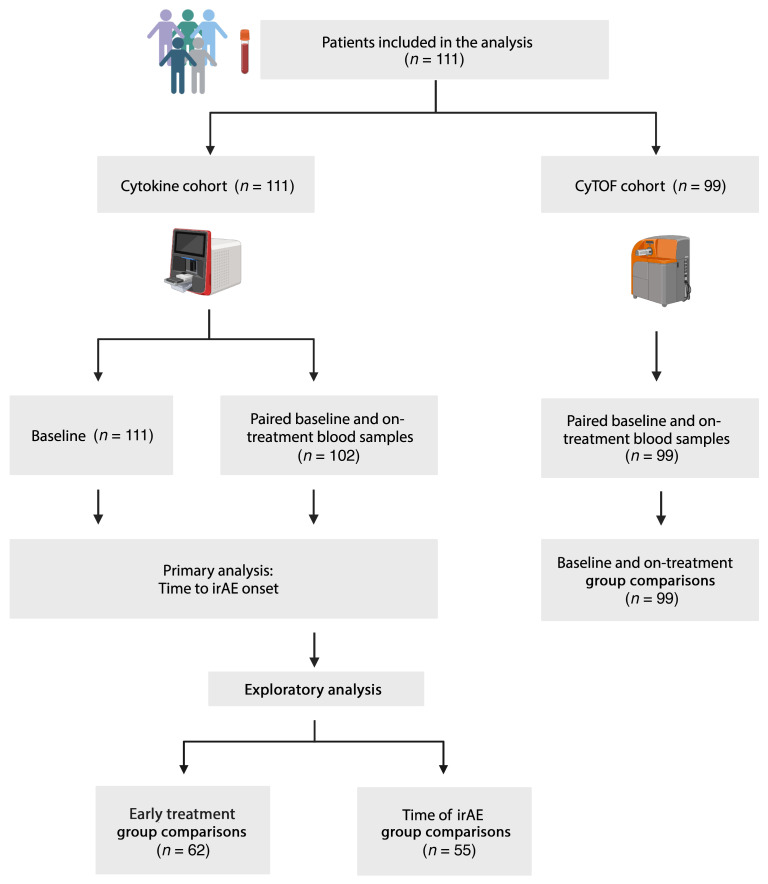
Study schema. Diagram showing an overview of the key downstream analyses. Further details are provided in the Methods and Supplemental Materials. Created using icons from BioRender.com.

From June 2021 to May 2024, we enrolled and performed analysis on 111 patients who received ICIs as standard of care at Johns Hopkins and prospectively followed them for up to 12 months from the last dose of ICI to monitor for irAE development in this observational study (Figure 1). The average follow up time from start of ICI was 7.4 months, and the max follow up time was 16.6 months. The baseline demographics, tumor types, treatment regimens, and clinical outcomes of the patients in our cohort are shown in Table 1 and Supplemental Table 1; supplemental material available online with this article; https://doi.org/10.1172/JCI176567DS1 Our cohort was comprised of 35.1% (*n* = 39) female patients and 64.9% male patients, and the median age of the cohort was 65 years (range 20 to > 90), and 31 patients identified as Black (27.9%), while 65.8% were white and 6.3% identified as “other” (see Supplemental Methods for detailed description). Most patients (*n* = 100, 90.1%) were treated in the advanced/metastatic setting. Reflecting patients eligible for ICI treatment as standard of care at Johns Hopkins, this cohort was diverse and heterogenous with a mixture of tumor types, ICI treatment regimens, and prior lines of cancer treatment. The most common tumor types were hepatocellular carcinoma (HCC) (*n* = 30, 27.0%) and renal cell carcinoma (RCC) (*n* = 24, 21.6%). All 111 included patients received anti-PD-1 or anti-PD-L1 therapy, and 23.4% (*n* = 26) received anti-PD-1 in combination with anti-CTLA-4 (*n* = 24) or anti-LAG-3 (*n* = 2). A history of prior oncologic systemic therapy was present in 41.4% of patients (*n* = 46), and 5.4% of patients (*n* = 6) had received prior ICI therapy before participating in the study. A baseline or preexisting autoimmune disorder was present in 12.6% (*n* = 14), and 4 out of these 14 patients were receiving systemic treatment with glucocorticoids or disease-modifying anti-rheumatic drugs (DMARDs) at the time of consent. Further details on autoimmune diseases present at baseline can be found in Supplemental Table 2. Grade ≥ 2 irAEs occurred in 45 patients (40.5%), and the distribution of grades can be found in Supplemental Table 1. ICI was permanently discontinued during the study period due to irAEs in 18.9% (*n* = 21) of patients, and, among the 45 patients who developed grade ≥ 2 irAEs, 62.2% (*n* = 28) received corticosteroids and 17.8% (*n* = 8) received immunosuppression. For the cytokine analysis, 111 patients had baseline samples, and 102 patients had an on-treatment sample, of which 88 had early on-treatment (month 1 or 2) samples and 24 had irAE samples. In the CyTOF cohort, a total of 99 patients had paired on-treatment and baseline samples.

**Figure 2:**
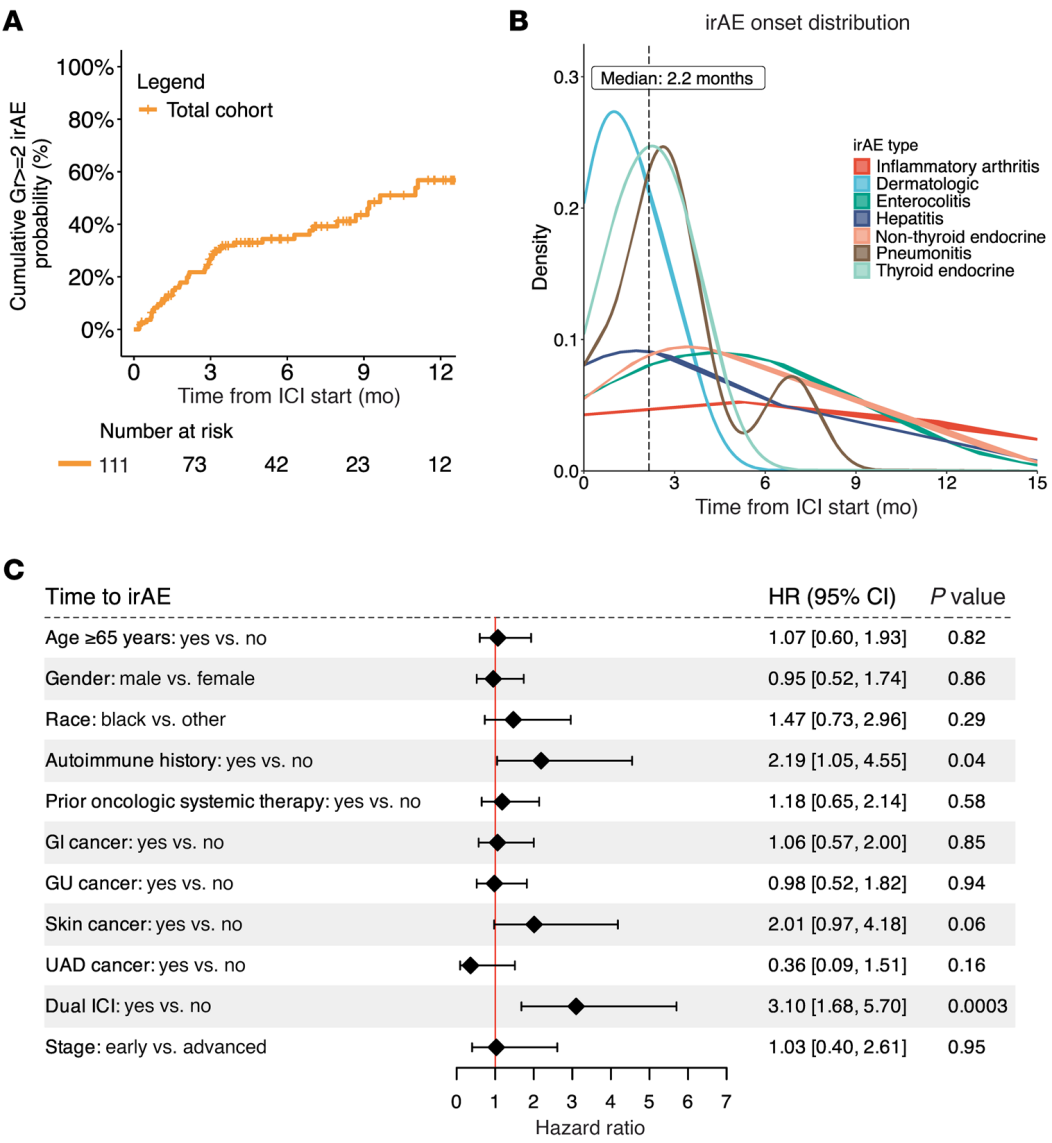
Grade ≥ 2 irAE distribution and association with clinicopathologic factors. (**A**) Cumulative probability of grade ≥ 2 irAEs in the total cohort (*n* = 111). (**B**) Density plot showing the onset of different types of grade ≥ 2 irAEs with at least 2 events in the cohort (*n* = 40). (**C**) Forest plot displaying the HRs for time to grade ≥ 2 irAE onset from a univariate Cox model (*n* = 111). CI, confidence interval; GI, gastrointestinal; Gr≥2, grade ≥ 2; GU, genitourinary; HR, hazard ratio; ICI, immune checkpoint inhibitor; UAD, upper aerodigestive.

Since grade 1 irAEs are mild, generally asymptomatic, and therapeutic interventions are usually not indicated, we focused on grade ≥ 2 irAEs as the main endpoint to enrich for clinically relevant irAEs and reduce confounding for patients who received ICI in combination with other therapies. The distribution and time course of grade ≥ 2 irAEs observed is shown in Table 2 and Figure 2, A and B. Of the 111 patients, 37 developed at least 1 grade ≥ 2 irAE within 6 months of ICI initiation (33.3%), and 12 developed at least 1 grade ≥ 2 irAE after the 6-month timepoint (10.8%). Thirteen patients (11.7%) developed 2 or more grade ≥ 2 irAEs, among whom 6 developed 2 grade ≥ 2 irAEs, 5 developed 3 grade ≥ 2 irAEs, and 2 developed 4 grade ≥ 2 irAEs. The most common grade ≥ 2 irAEs within 6 months of ICI initiation included 16 cases of dermatologic irAEs, 12 cases of hypothyroidism, 5 cases of enterocolitis, and 5 cases of pneumonitis.

Among the key baseline clinicopathologic factors, the use of combination ICI therapy (hazard ratio [HR] 3.10, 95% confidence interval [CI] 1.68–5.70, *P* = 0.0003) and baseline autoimmune history (HR 2.19, 95% CI 1.05–4.55, *P* = 0.04) were associated with an increased risk of grade ≥ 2 irAE, while the other clinicopathologic factors, including reported race, were not significant (Figure 2C). These observations confirm the increased risk and frequency of irAEs with combination ICI therapy and baseline autoimmune history and the importance of addressing these as potential confounders.

### Baseline cytokines are not predictive for irAEs.

In total, 111 patients had quantification of plasma cytokine levels of pretreatment samples to assess for baseline predictors of grade ≥ 2 irAEs. A 32-plex cytokine assay was used for high-throughput investigation. We observed relevant baseline heterogeneity in pretreatment cytokine levels based on selected clinically important characteristics. Patients with baseline autoimmune history had significantly higher levels of IP-10, MCP-1, MIG, and RANTES (Supplemental Figure 1; *P* < 0.05). IL-17a, IL-17f, IL-4, sCD40L, and VEGF-A plasma levels were significantly lower in patients who had previously received oncologic systemic therapy (Supplemental Figure 2; *P* < 0.05). Cancer histology grouped into gastrointestinal (GI), genitourinary (GU), upper aerodigestive (UAD), skin, and other showed differences in plasma levels of IL-8, IL-17f, IP-10, and RANTES at baseline (Supplemental Figure 3; *P* < 0.05). Among the 32 cytokines analyzed, there were no cytokines predictive of grade ≥ 2 irAEs based on the time to irAE analysis after multitesting adjustment in both the unadjusted and adjusted Cox model, accounting for baseline autoimmune history and prior systemic therapy (Supplemental Table 3). Due to this baseline heterogeneity, all subsequent analyses involving plasma cytokines and on-treatment samples were performed with fold change. Apart from the pretreatment heterogeneity, our data were consistent with prior reports that any predictive value of pretreatment cytokine signatures for irAE development is likely tumor dependent, while there is no unifying cytokine signature predictive across tumor types (9–12). Published studies on baseline blood cytokines have primarily explored these associations in melanoma and non-small cell lung cancer (NSCLC) and found mixed results with various cytokines and chemokines implicated in irAE development (9-12).

### Early changes in T helper-related cytokines after ICI initiation precede irAE development.

**Figure 3:**
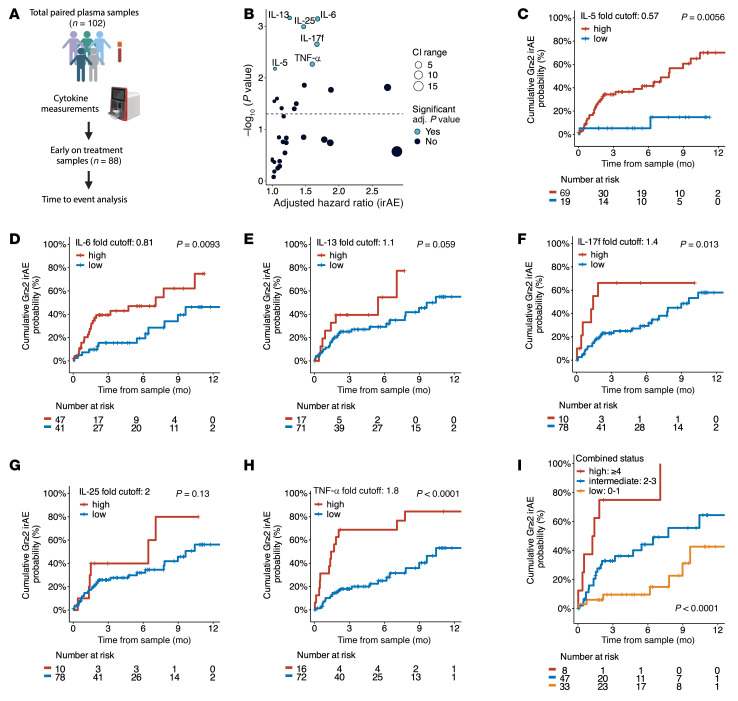
Early changes in T helper–associated cytokines precede grade ≥ 2 irAE development. (**A**) Schema for early on-treatment time event analysis (*n* = 88). (**B**) Scatterplot displaying the adjusted HR for early on-treatment cytokine fold change and time to onset of grade ≥ 2 irAEs utilizing a multivariate Cox model and multitesting adjustment. The dotted line represents unadjusted *P* = 0.05, in which cytokines above the line are significant without multitesting correction. The size of each cytokine dot represents the width of the 95% CI range. (**C**–**H**) Reverse Kaplan-Meier (KM) plots for cumulative probability of grade ≥ 2 irAEs for statistically significant cytokines from the multivariate Cox model after multitesting adjustment. Optimal cutoffs were determined using maximally selected log-rank statistics. (**I**) Reverse KM plot for the cumulative probability of grade ≥ 2 irAEs utilizing a combined cytokine status based on presence of high cytokine fold changes as determined by the optimal cutoffs calculated for the 6 cytokines: low, 0-1 cytokines; intermediate, 2-3 cytokines; and high, ≥4 cytokines. Time to irAE onset or last follow up was adjusted for time to early on-treatment sample collection to account for immortal time bias. adj., adjusted; CI, confidence interval; Gr≥2, grade ≥2; HR, hazard ratio; KM, Kaplan Meier.

Next, we sought to investigate whether early treatment-related changes in plasma cytokines were predictive of grade ≥ 2 irAEs in the 88 patients with available early on-treatment samples (Figure 3A and Supplemental Table 4). Early on-treatment analyses included only patients with paired month 1 or 2 on-treatment and baseline samples and excluded patients who developed grade ≥ 2 irAEs prior to collection of early on-treatment plasma. The median time from ICI initiation to early on-treatment sample collection was 1.38 months, and the median time from collection of the early on-treatment sample to the grade ≥ 2 irAE was 1.53 months. Given the heterogeneity of our cohort including survival time, we adjusted our time-to-event Cox models for age, sex, race, treatment with dual ICI, prior oncologic treatment, and disease stage. Three plasma cytokine signatures conferred an increased risk of grade ≥ 2 irAE development: IL-5, IL-13, and IL-25 (type 2), IL-6 and IL-17f (Th17 related), and TNF-α (type 1) (Figure 3B and Table 3; *P*_adj_ < 0.05). In light of the observed clinical association between dual ICI use and risk of irAEs, we performed an exploratory subgroup analysis to investigate whether the cytokine signatures across the entire cohort were driven by use of dual ICI, using a multivariate Cox model that also adjusted for age, gender, race, prior oncologic treatment, and disease stage. Among the 88 patients with available early on-treatment samples, 38.9% of patients treated with dual ICI (*n* = 7 of 18) and 32.9% of single ICI patients (*n* = 23 of 70), which included ICI monotherapy or a single ICI in combination with chemotherapy or targeted therapy, developed a grade ≥ 2 irAE. No early fold changes in cytokines met statistical significance for time to onset of grade ≥ 2 irAEs after multi-testing adjustment for the dual ICI cohort, while all 6 significant cytokines (IL-5, IL-6, IL-13, IL-17f, IL-25, and TNF-α) from the multivariate Cox model for the total early on-treatment cohort were still significant for the single ICI cohort with the addition of IL-1α, IL-2, IL-9, IL-18, IL-22, MCP-1, and MIP-1β (Supplemental Tables 5 and 6, *P*_adj_ < 0.05). Though limited by power, these findings provided support that the observed cytokine signatures in the total early on-treatment cohort were not disproportionately driven by dual ICI treatment. It is known that type 2 cytokines produced by Th2 cells can drive B cell differentiation and eosinophil recruitment, both immunopathogenic drivers of allergic and atopic diseases, while Th17 cytokines including IL-6 and IL-17 are critical mediators for several autoimmune diseases including rheumatoid arthritis and psoriasis (23, 33–35). Though traditionally associated with Th1 cells and a type 1 cytokine response, TNF-α has also been implicated in the differentiation of Th17 cells and promotion of IL-17 secretion by Th1 and Th17 cells in autoimmune disease (36–38). Our observation suggests that these distinct cytokine pathways, which are key pathogenic drivers in some common autoimmune diseases, may also be critical to the development of irAEs.

To visualize the time to event analyses, we calculated optimal cutoffs using the maximally selected log-rank method to classify high and low levels of the significant cytokines selected from the multivariate Cox model (IL-5, IL-6, IL-13, IL-17f, IL-25, and TNF-α) for the total early on-treatment cohort (39). Of these 6 cytokines, IL-5, IL-6, IL-17f, and TNF-α dichotomized patients at a higher risk for grade ≥ 2 irAEs by an optimal cutoff of 0.57, 0.81, 1.4, and 1.8 fold, respectively (Figure 3, C–H; *P* < 0.05). Individually, IL-13 and IL-25 did not significantly stratify patients for grade ≥ 2 irAEs based on the calculated optimal cutoff, which may be due to loss of power with a binary categorization, nominalizing continuous data into nonbiological categories, or may also reflect their importance in a smaller subset of irAEs. Nonetheless, the optimal cutoffs of each individual cytokine were selective for distinct patient subsets and suggested synergism if combined.

To explore whether combining high plasma cytokine status could improve risk stratification, we classified patients based on the presence of high cytokine levels for these 6 cytokines: low, 0–1 cytokines; intermediate, 2–3 cytokines; and high, ≥4 cytokines. The risk of developing grade ≥ 2 irAEs was found to be the highest in cases when ≥ 4 cytokines were elevated and sequentially had lower risk with decreasing number of high cytokines (Figure 3I; *P* < 0.0001). Confirmation of the risk classification ability of these cytokines is outside the scope of the current study and would require validation beyond our single institution.

**Figure 4:**
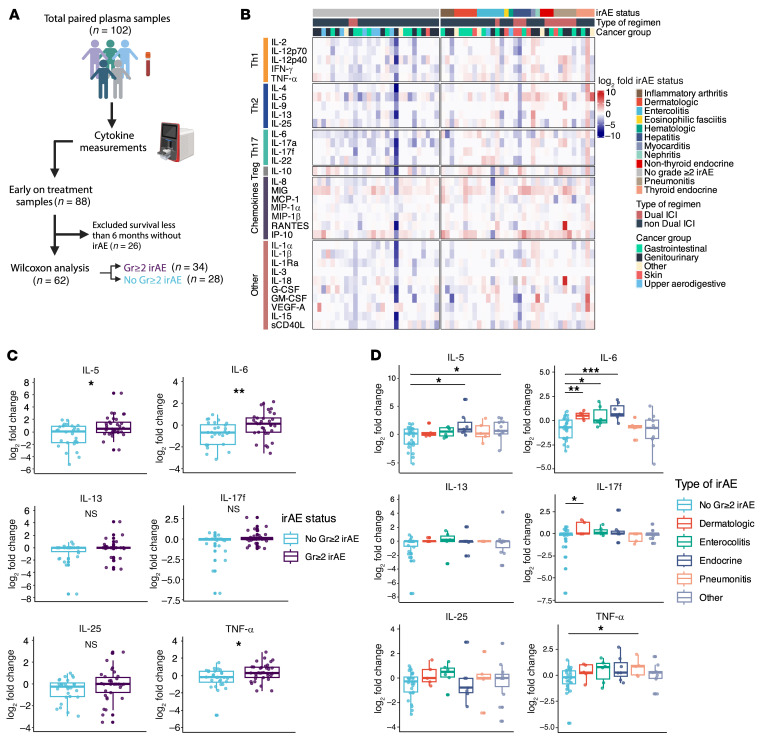
Early treatment changes in T helper associated cytokines are associated with distinct grade ≥ 2 irAEs. (**A**) Schema for early on-treatment cytokines and irAE group comparisons (no grade ≥ 2 irAE, *n* = 28; grade ≥ 2 irAE, *n* = 34). (**B**) Heatmap showing early treatment fold changes of 32 cytokines grouped by future irAE status. Additional clinical annotations include the type of ICI regimen given and cancer group. (**C**) Boxplots showing log_2_ transformed cytokine fold changes between patients who develop future grade ≥ 2 irAE or not. (**D**) Boxplots showing log_2_ transformed cytokine fold changes between patients who develop specific types of irAEs compared with no grade ≥ 2 irAE. Comparisons between groups were performed using Wilcoxon rank-sum test. Abbreviations: Gr≥2, grade ≥ 2; ICI, immune checkpoint inhibitor. *P < 0.05, **P < 0.01, ***P < 0.001.

Because these cytokine pathways are known to drive different autoimmune disease phenotypes, we investigated whether these early on-treatment cytokine signatures were associated with distinct organ-specific grade ≥ 2 irAEs (Figure 4 and Supplemental Tables 7 and 8). To account for survivorship bias and enrich for patients with adequate time to develop irAEs in group comparisons involving irAE status, patients were considered not to have developed grade ≥ 2 irAEs only if they had at least 6 months of follow up without evidence of toxicity (Figure 4A). We observed a significantly higher fold change in IL-5, IL-6, and TNF-α between patients with grade ≥ 2 (*n* = 34) and no grade ≥ 2 irAEs (*n* = 28) (Figure 4, B and C; *P* < 0.05). This observed difference was driven primarily by endocrine (*n* = 7) and other irAEs (*n* = 11) for IL-5; dermatologic (*n* = 5), enterocolitis (*n* = 6), and endocrine irAEs for IL-6; and pneumonitis (*n* = 5) for TNF-α (Figure 4D; *P* < 0.05). Though fold change differences in IL-17f were not significant for all grade ≥ 2 irAEs, IL-17f was increased in dermatologic irAEs (Figure 4, C and D; *P* < 0.05). IL-13 and IL-25 were not significantly associated with any specific irAE but showed a trend toward higher fold change for enterocolitis irAEs (Figure 4, C and D; *P* = 0.07 and *P* = 0.05, respectively). Overall, these data imply that early changes in T helper related cytokine signatures can indicate the development of grade ≥ 2 irAEs and may have independent associations with distinct types of irAEs.

### IL-6 is associated with both an increased irAE risk and inferior cancer-related outcomes.

Post hoc analyses of prior ICI interventional trials and retrospective studies have collectively identified a modest but reproducible correlation between antitumor responses and irAEs (40–43). Although the cumulative risk of irAEs increases with greater time on ICI therapy, such correlations remain even in patients who have early discontinuation due to toxicity (40–43). This correlation between efficacy and toxicity has been observed in different tumor types and appears somewhat dependent on the severity of irAEs with improved survival in low grade irAEs (43–45). However, many patients achieving clinical responses to ICIs do not experience toxicity; conversely, many patients experiencing irAEs do not achieve clinical responses to ICI therapy (7). This partial uncoupling of benefit and toxicity from ICIs provides initial clinical evidence that mechanistic drivers of irAEs may be distinct from pathways that drive antitumor immunity.

**Figure 5:**
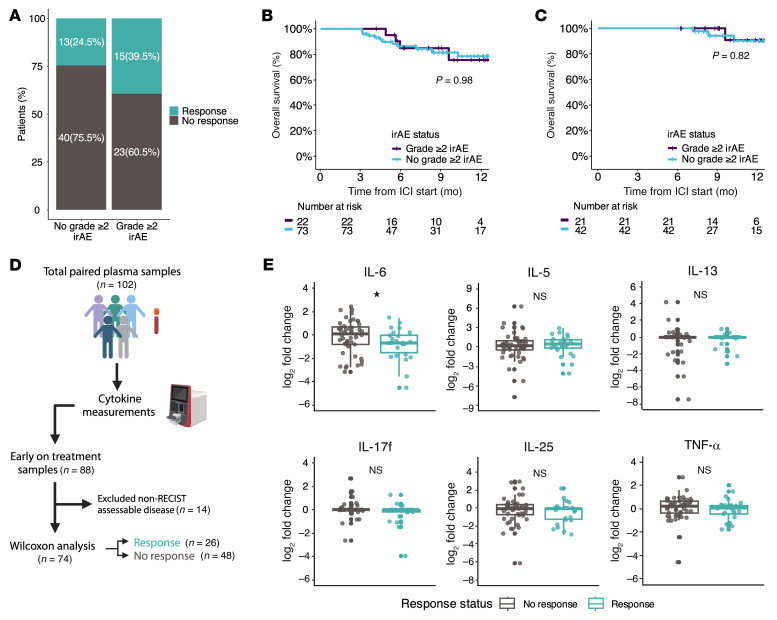
Higher early treatment changes in IL-6 are associated with nonresponse. Objective response was defined as complete response or partial response, while nonresponse included stable disease and progression. (**A**) Stacked barplot showing difference in best objective response between patients who develop future grade ≥ 2 irAE or not (91 patients had RECIST assessable disease). Fischer’s exact test was nonsignificant, *P* = 0.17. Landmark analysis at (**B**) 3 months (*n* = 95) and (**C**) 6 months (*n* = 63) to assess the influence of grade ≥ 2 irAE development on survival. For landmark analyses, grade ≥ 2 irAEs had to occur prior to the landmark time, and significance testing was performed with log-rank test. (**D**) Schema for early on-treatment cytokines and response comparisons. (**E**) Boxplots showing early treatment log_2_ transformed cytokine fold changes between patients who achieved an objective response (*n* = 26) or not (*n* = 48). Comparisons between groups were performed using Wilcoxon rank-sum test. Abbreviations: Gr≥, grade ≥ 2. *P < 0.05.

**Figure 6:**
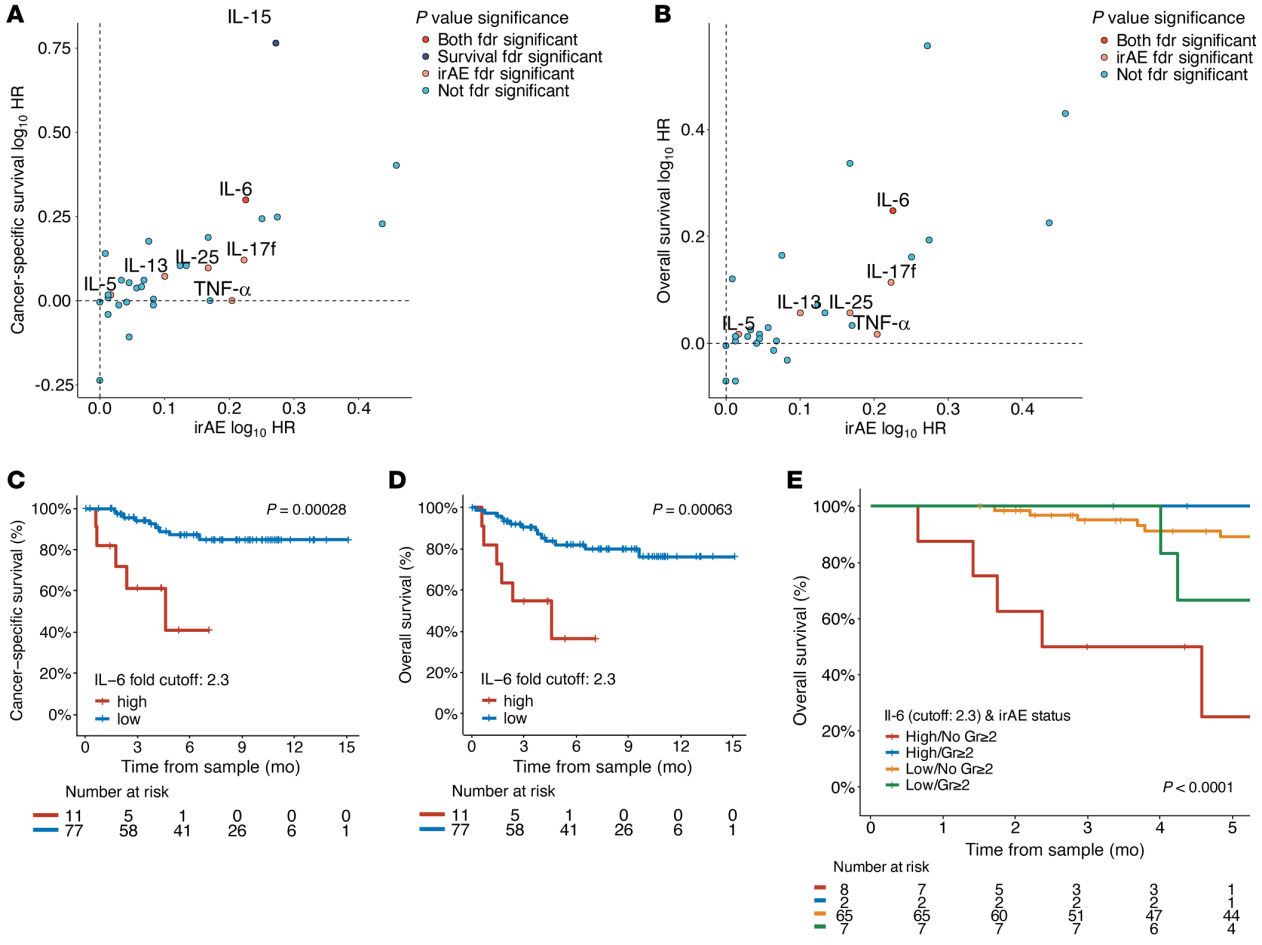
Higher early treatment changes in IL-6 are associated with worse cancer-specific and overall survival. From the multivariate Cox models for the early on-treatment cohort (*n* = 88), scatterplots displaying log_10_ transformed adjusted HRs for early on-treatment cytokine fold changes and the time to grade ≥ 2 irAE onset compared with (**A**) cancer-specific survival and (**B**) overall survival. Cytokines that are significant after FDR adjustment are displayed. (**C**) Cancer-specific survival and (**D**) overall survival KM curves stratified by an optimal fold change cutoff of 2.3 for early treatment changes in IL-6. The optimal cutoff was determined using maximally selected log-rank statistics. (**E**) Landmark analysis at 10 weeks stratified by optimal fold change cutoff of 2.3 for early treatment changes in IL-6 and grade ≥ 2 irAE development. For landmark analyses, grade ≥ 2 irAEs had to occur prior to the landmark time. Significance for KM curves was assessed utilizing log-rank test. FDR, false discovery rate; Gr≥2, grade ≥ 2; HR, hazard ratio; KM, Kaplan Meier.

Across the present cohort, among patients with measurable disease, 30.8% (28 of 91) patients achieved a best response of partial or complete response (objective response) to ICI therapy by RECIST 1.1 criteria. Nonresponse was defined as progression or stable disease, and this best radiographic response was determined utilizing RECIST 1.1 criteria from imaging ordered by the primary oncologist from baseline, prior to ICI initiation, to the last scan before censorship (additional details in the Extended Methods of the Supplemental Materials). There was no significant association between grade ≥ 2 irAEs and objective response rate (ORR) with ICI therapy (ORR 39.5% in grade ≥ 2 irAEs versus 24.5% in no grade ≥ 2 irAEs by Fisher’s exact test, *P* = 0.17) (Figure 5A). Further, patients who experienced a grade ≥ 2 irAE had a similar overall survival compared with patients who had no grade ≥ 2 irAEs at landmarks of 3 and 6 months (Figure 5, B and C; *P* = 0.98 and *P* = 0.82, respectively). Among the 6 cytokines associated with grade ≥ 2 irAEs, higher early fold change in IL-6 was associated with worse tumor responses (Figure 5, D and E; *P* = 0.048), while the other 3 cytokines were not associated with tumor responses by RECIST 1.1 (Figure 5, D and E and Supplemental Table 9). Early plasma cytokine increases in IL-6 levels were also associated with both cancer-specific and all-cause mortality. Among all 32 cytokines quantified, only early on-treatment fold changes of IL-6 was significantly associated with increased risk of grade ≥ 2 irAEs and both cancer-specific and all-cause mortality after multitesting adjustment (Figure 6, A and B and Supplemental Tables 10 and 11). Given the diversity of tumor types and inclusion of patients treated in the second-line and beyond, the lack of cytokine associations with response is not unexpected, and additional evaluations in more homogenous populations are needed. Nonetheless, our findings with IL-6 provide support for the partial uncoupling of drivers of antitumor immunity and irAE-related autoimmunity.

The relationship between IL-6 and inferior cancer-related outcomes in this diverse, pan-tumor cohort are congruent with prior retrospective analyses of interventional clinical trials and observational studies from single tumor types (46–48). Several preclinical studies have shown significantly improved tumor control and survival with combination IL-6 blockade (anti-IL6 or anti-IL-6R) and ICI compared with ICI alone, which was postulated to be due to improved Th1/Th17 skewing (49–53). Recently, upfront IL-6 blockade mitigated irAE symptoms in a murine model of experimental autoimmune encephalomyelitis while preserving antitumor activity after anti-CTLA-4 exposure (53). Our results support these observations, and we sought to further understand the role of IL-6 in the spectrum of irAE and clinical benefit. Unlike for irAEs, IL-6 significantly dichotomized patients at increased risk for cancer-specific and all-cause mortality at a much higher optimal cutoff of 2.3 fold change (Figure 6, C and D; *P* < 0.001), revealing that the subset of patients with the highest increase in IL-6 was at a disparate risk of early death. To assess whether the increased mortality with higher IL-6 fold change was due to the development of grade ≥ 2 irAEs, we used a 10-week landmark analysis to capture the effect of early irAEs on overall survival. Of note, due to our limited cohort size after landmark classification, we could not assess the effect of late onset irAEs (≥ 6 months). In the 10-week landmark, patients with high early IL-6 increase (≥ 2.3 fold) without grade ≥ 2 irAEs had the worst overall survival; in contrast, if patients with high IL-6 also developed a grade ≥ 2 irAE by 10 weeks, then the survival curves were in line with patients with low IL-6 (Figure 6E; *P* < 0.0001). Taken together, these data suggest that there appears to be a spectrum of toxicity and ICI resistance that is dependent on the degree of early treatment IL-6 changes. We believe that this knowledge could be therapeutically exploited by concurrent upfront or early inhibition of IL-6 pathways in combination with ICIs for both antitumor effect and prophylaxis for patients at high risk of irAEs.

### Th17 cytokines are persistently higher during the acute phase of irAEs.

**Figure 7:**
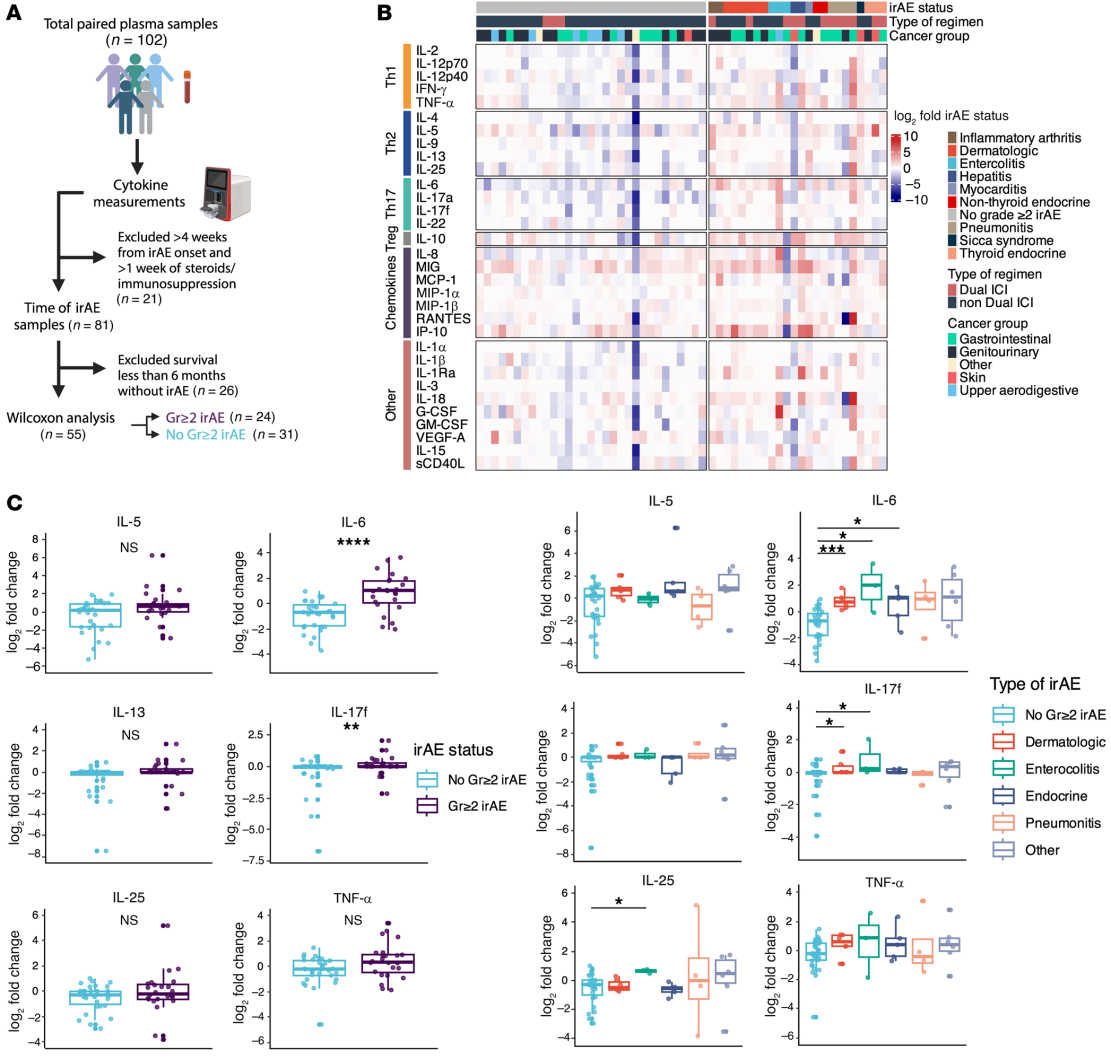
Higher changes in Th17 cytokines are detected at the onset of grade ≥ 2 irAEs. (**A**) Schema for time of irAE cytokines and irAE group comparisons. (**B**) Heatmap showing on-treatment fold changes of 32 cytokines grouped by irAE status (no grade ≥ 2 irAE, *n* = 31; grade ≥ 2 irAE, *n* = 24) near the time of irAE onset. Additional clinical annotations include the type of ICI regimen given and cancer group. (**C**) Boxplots showing log_2_ transformed cytokine fold changes between patients who developed a grade ≥ 2 irAE or not near the time of irAE onset. (**D**) Boxplots showing log_2_ transformed cytokine fold changes between patients who develop specific types of irAEs compared with no grade ≥ 2 irAE near the time of irAE onset. Comparisons between groups were performed using Wilcoxon rank-sum test. Gr≥2, grade ≥2; ICI, immune checkpoint inhibitor. *P < 0.05, **P < 0.01, ***P < 0.001, ****P < 0.0001.

Our findings indicate the importance of early changes in type 1, type 2, and Th17 cytokines and the development of grade ≥ 2 irAEs. Thus, we sought to confirm whether these cytokines continued to be differentially expressed at the time of irAE for grade ≥ 2 irAEs (*n* = 24) compared with the on-treatment timepoint for patients with no grade ≥ 2 irAEs (*n* = 31) (Figure 7, A and B and Supplemental Tables 12 and 13). The median time after ICI initiation for collection of irAE samples for patients with grade ≥ 2 irAEs (1.4 months, range 0.2–9.2 months) was not different compared with on-treatment samples for patients with no grade ≥ 2 irAE utilized in this analysis (1.5 months, range 1.3–12.2 months) (*P* = 0.44), confirming that timing of sample collection was not significantly different between groups. Among Th17 related cytokines, IL-6 and IL-17f were significantly increased in grade ≥ 2 irAEs compared with no grade ≥ 2 irAEs (Figure 7C; *P* = 0.00001 and *P* = 0.01, respectively), while TNF-α trended toward significantly increased in grade ≥ 2 irAEs (Figure 7C; *P* = 0.06). The Th2-related cytokines (IL-5, IL-13, and IL-25) were not significantly different, though IL-5 was trending toward increased levels in grade ≥ 2 irAEs (Figure 7C; *P* = 0.07). IL-6 was primarily elevated in dermatologic (*n* = 6, *P* = 0.0003), enterocolitis (*n* = 3, *P* = 0.01), and endocrine (*n* = 5, *P* = 0.03) irAEs (Figure 7D). IL-17f was associated with both dermatologic and enterocolitis irAEs, while IL-25 was elevated in enterocolitis irAEs (Figure 7D; *P* < 0.05). IL-5 and IL-13 did not have significant organ-specific irAEs, but IL-5 was trending toward significantly higher levels in endocrine (*n* = 5, *P* = 0.07) and other irAEs (*n* = 6, *P* = 0.07) (Figure 7D). Despite limited power due to the small number of organ-specific irAEs, we still detected a strong signal for IL-6 as a key mediator of irAEs and support for the importance of Th17 cytokines during the acute phase of irAEs.

### Expansion of Th17 and Th2 populations in patients with irAEs.

**Figure 8:**
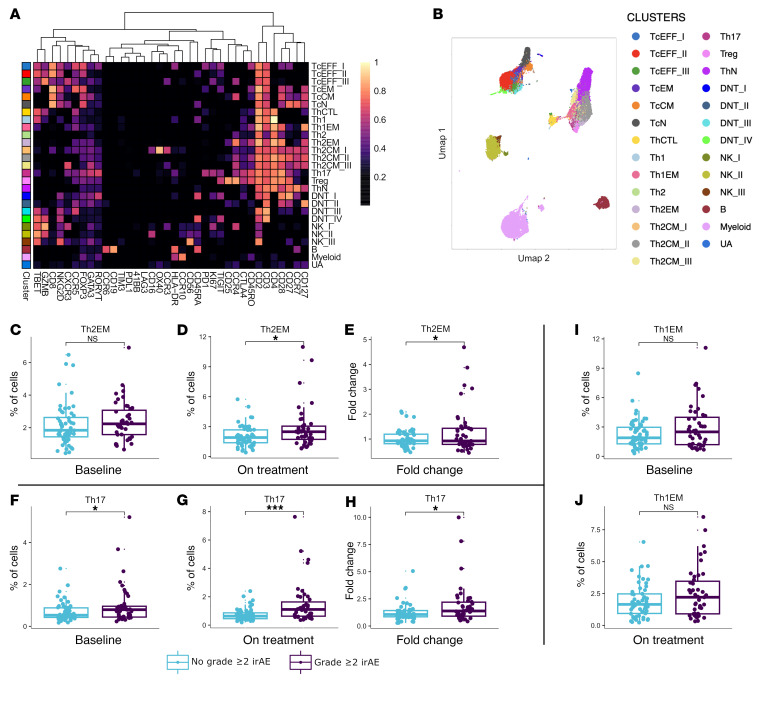
Higher fold change in abundance of Th17 and Th2EM cells are associated with the development of grade ≥ 2 irAEs. CyTOF analysis of 99 paired on-treatment and baseline PBMC samples to assess cellular differences based on grade ≥ 2 irAE status. On-treatment PBMC samples utilized in this analysis included the closest sample to onset of the grade ≥ 2 irAE or the earliest available timepoint for patients without grade ≥ 2 irAEs. PBMC samples were thawed and assayed by a 37-marker CyTOF panel. A FlowSOM algorithm was used to generate 40 metaclusters, which were annotated into a final 27 clusters. (**A**) Scaled expression profile for each cluster is shown in the heatmap. (**B**) UMAP plots visualizing the annotated clusters (200 cells per sample). Boxplots showing the abundance of Th2EM cells at (**C**) baseline, (**D**) on treatment, and (**E**) fold change between the 2 timepoints in patients with grade ≥ 2 irAEs (*n* = 43) or not (*n* = 56). Boxplots showing the abundance of Th17 cells at (**F**) baseline, (**G**) on treatment, and (**H**) fold change between the 2 timepoints. Boxplots showing the abundance of Th1EM at (**I**) baseline and (**J**) on treatment. Comparisons between groups were performed using unpaired *t* test. CTL, cytotoxic T lymphocyte; CM, central memory; DNT, double-negative T; EFF, effector; EM, effector memory; *N*, naive; NK, natural killer; Tc, cytotoxic T cell; Th, helper T cell; UA, unassigned. *P < 0.05, ***P < 0.001.

We next sought to characterize peripheral blood immune cell populations that drive grade ≥ 2 irAEs, and specifically to determine whether changes in circulating Th17, Th2, and Th1 immune populations mirrored our observed changes in peripheral cytokines. We utilized pre- and on-treatment peripheral blood mononuclear cells (PBMCs) using a 37-antibody panel by CyTOF (Supplemental Table 14). On-treatment PBMC samples utilized in this analysis included the closest sample to onset of the grade ≥ 2 irAE or the earliest available on-treatment timepoint for patients without grade ≥ 2 irAEs (additional details for sample selection can be found in Data Analysis Overview in the Methods). On-treatment samples ranged from 0.2 months to 9.2 months with a median of 1.3 months after ICI initiation. This robust CyTOF panel allowed us to assess treatment-induced changes in the type-1 and type-2, versus type-17 phenotypes (CXCR3, CCR4, CCR5, CCR6, GATA3, RORγ, and T-bet), Tregs (FOXP3), along with abundance of naive states (CD45RA, CCR7, and CD45RO) and the promotion of T cell activation/exhaustion (GZMB, KI67, PD1, 41BB, and LAG3) (Supplemental Table 15). Our CyTOF cohort consisted of 99 patients (characteristics in Supplemental Table 16), and the CyTOF data acquisition, processing, and normalization pipeline is detailed in Extended Methods and Supplemental Figure 4. As shown on the expression profiles of the annotated cluster heatmap and the accompanying UMAP visualization of unsupervised clustering results, there were a variety of CD3^+^CD4^+^ helper T cell subtypes within the resulting data set, namely several Th1, Th2, and Th17 clusters (Figure 8, A and B and Supplemental Table 15).

Among the Th2 clusters, there were no significant differences in the abundance of Th2 effector memory (Th2EM) cells at baseline in patients who developed grade ≥ 2 irAEs (*n* = 43) compared with patients who did not (*n* = 56) (Figure 8C and Supplemental Table 17, *P* = 0.37), which was consistent with our baseline cytokine results. However, after ICI treatment, we observed propagation of the Th2 response as evidenced by both a higher abundance (Figure 8D; *P* = 0.01) and concurrent expansion of Th2EM cells (Figure 8E; *P* = 0.03) in patients who developed grade ≥ 2 irAEs (Supplemental Tables 18 and 19).

Unlike Th2EM cells, the abundances of Th17 cells were significantly different at baseline in patients with grade ≥ 2 irAEs compared with those without (Figure 8F and Supplemental Table 17; *P* = 0.03). After ICI exposure, this difference became even more pronounced with persistently higher abundances (Figure 8G; *P* < 0.001) and increased expansion of Th17 cells (Figure 8H; *P* = 0.02) in patients who develop grade ≥ 2 irAEs (Supplemental Tables 18 and 19). Though we did not observe a statistically significant difference in immune clusters associated with type 1 responses, a Th1EM subset trended toward higher abundance at baseline and on treatment in patients who develop grade ≥ 2 irAEs (Figure 8, I and J; *P* = 0.06 for both). Overall, these data are broadly congruent with our cytokine data, showing a relationship between increases in both Th2EM and Th17 cell subsets and irAEs with ICI therapy.

## Discussion

As the utilization of ICIs increases, particularly in patients with early stage cancers who are at risk for long-term toxicities of cancer therapy, irAEs are an increasing concern in clinical practice. There is a critical need to develop effective interception and treatment strategies for irAEs, particularly with therapeutic strategies that do not compromise antitumor immunity. Through our integrated clinical-biomarker–immunologic analyses derived from a diverse, pan-tumor cohort, we found that early increases in Th17 (IL-6, IL-17f), type 2 (IL-5, IL-13, IL-25), and type 1 (TNF-α) cytokines were associated with the development of grade ≥ 2 irAEs. Among these cytokines, the Th17-related cytokine, IL-6 and IL-17f showed the strongest association with grade ≥ 2 irAEs both in early on treatment and at the time of irAE. Similarly, we find that the abundances of Th17 cells at treatment baseline and early on-treatment expansions of Th17 and Th2EM T cell populations are positively associated with the development of grade ≥ 2 irAEs.

The cytokines and cellular populations linked to irAEs in the current analysis have been extensively investigated in the context of spontaneous autoimmunity (21–23, 25–27). For the type 2 pathway, IL-25, a type 2 alarmin, promotes Th2 differentiation and secretion of IL-4, IL-5, and IL-13, which are important for eosinophil differentiation (33, 54–58). This type 2 response is the pathogenic driver of numerous diseases including asthma, atopic dermatitis, inflammatory lung disease, eosinophilic GI diseases, and lupus nephritis (21, 33, 59, 60). In regards to the Th17 pathway, IL-6 promotes the differentiation of naive T cells to Th17 and expression of IL-17 through activation of the transcription factor ROR-γτ, resulting in expression of IL-23 that enhances stabilization of the Th17 phenotype by promoting IL-17 and IL-22 expression (35, 61). Lastly, TNF-α both promotes and inhibits inflammation through signaling through TNFR1 and TNFR2, and the differential signaling through these receptors on various types of cells, including Th1 and Th17 cells, can lead to the pathogenesis of various autoimmune diseases (27). IL-5, IL-6, IL-13, IL-17, IL-23, and TNF-α are targetable with biologics developed for spontaneous autoimmunity, and our data broadly support the prospective investigation of these inhibitors for patients with irAEs (21–23, 26, 27, 62).

Our data builds upon prior studies of drivers of irAEs, including analyses of cytokine and immune subsets primarily in melanoma and NSCLC. In melanoma, low IL-6 or high IL-17 at baseline were associated with severe irAEs following anti-CTLA-4 blockade while an aggregated CYTOX score consisting of 12 cytokines and chemokines, including IL-13, had a modest predictive performance (AUC = 0.68–0.70) for severe irAEs in patients treated with anti-PD-1 alone or in combination with anti-CTLA-4 inhibitors (9, 63, 64). In cohorts comprising primarily lung cancers, high baseline and upregulation of IL-10 after ICI treatment, lower baseline levels of various chemokines (CXCL9, CXCL10, CXCL11, and CXCL19), increases in CXCL9 and CXCL10 after ICI exposure, and increased TNF were associated with the development of irAEs (10, 12, 65). These differences in results may be explained by the distribution of specific types of irAEs as some studies have shown distinct cytokine associations with organ-specific irAEs, such as IL-6 with colitis; IL-17 with colitis, thyroiditis, and pneumonitis; and IL-1β, IL-2, and GM-CSF with thyroid irAEs (11, 66–68). When investigating individual types of irAEs, Th17 cells and cytokines have also been observed in the peripheral blood or inflamed tissues of specific irAEs, including synovial fluid from inflammatory arthritis, colonic tissue in enterocolitis, and blood in psoriatic dermatitis and colitis irAEs (29, 53, 69, 70). Similarly, TNF-α has been increased in the synovial biopsies of ICI-induced inflammatory arthritis, peripheral blood, and upregulated on various cells including T cells and macrophages in irAEs (65, 71, 72). In support of distinct T cell populations driving irAEs, within a cohort of thymic and lung cancers, 4 distinct subtypes of T cells were associated with irAEs: Th17 related, TNF related, and 2 Treg clusters (28). Our study not only linked Th17 and TNF pathways to irAEs but provided support for investigation into Th2 and type 2 cytokines in the development of irAEs, which have not been explored as extensively. Some studies have shown peripheral eosinophil counts have been associated with an increased risk of irAEs; however, the link with specific type 2 cytokines has not been directly made (73, 74). Our type 2 cytokine signal was most robustly observed utilizing time-to-event analysis, which suggests there may be a loss of power when not incorporating follow up time and that timing of the sample collection may be especially important for this pathway. Overall, our data reaffirms the importance of the Th17 and TNF pathway in the development of irAEs, supports further investigation into Th2 clusters and type 2 cytokines, and provides evidence of differential cytokine expression in distinct types of irAEs in a prospective, pan tumor analysis.

A key consideration when treating irAEs in patients with cancer is the preservation of antitumor immunity, but the relationship between irAEs and antitumor immunity is not fully understood. Preclinical studies suggest that TNF inhibition may provide a dual benefit of improved tumor control and reduced toxicity (75, 76). Similarly, high IL-6 has been shown to be a poor prognostic marker to ICI response in multiple tumor cohorts, and preclinical data suggest a potential dual benefit of IL-6 blockade, including improved tumor control and reduced toxicity (46–53, 77, 78). These data led to the phase 1b clinical trial TICIMEL, assessing upfront TNF inhibition with dual ICI in advanced melanoma, as well as trials evaluating upfront tocilizumab, an anti-IL-6R antibody, in combination with ipilimumab and nivolumab in melanoma (NCT03999749) and in melanoma, NSCLC, or urothelial carcinoma (NCT04940299) (46–53, 77, 79). The relationship between the IL-17 pathway and antitumor immunity has been controversial and may be context dependent (80–83). Inhibition of the IL-17 pathway with secukinumab for the treatment of irAEs was associated with tumor progression in a single patient report, but other reports indicated successful irAE treatment without concurrent tumor growth (81, 84). A recent evaluation of melanoma patients receiving combination anti-CTLA-4 and anti-PD-1 found that increased IL-17a/IL-17f signaling was positively associated with immune infiltration and improved clinical outcomes; however, this relationship did not extend to patients treated with monotherapy (82). Therefore, the safety of treating irAEs with an IL-17 inhibitor may depend upon the ICI regimen. Although our study was underpowered to evaluate immunological changes of any specific checkpoint inhibitor, we observed similar cytokine signatures for the single-ICI group as the overall cohort, suggesting that the findings are not restricted to the smaller subset of patients receiving a dual-ICI treatment. In terms of the type 2 cytokines, prior data has also been conflicting about the relationship between IL-5, IL-13, and IL-25 and antitumor immunity with both evidence of promotion of metastasis or response to ICI (59, 85–90). However, recently, a preclinical study demonstrated that blockade of IL-25 through anti-IL-17RA reduced off target organ infiltration with immune cells and improved antitumor activity, and a case series showed high IL-4 and IL-13 in skin lesions of ICI induced bullous pemphigoid, which responded to dupilumab treatment (91, 92). In our analysis, no cytokine was associated with favorable clinical outcomes, and high IL-6 played a dual role in both irAEs and resistance to ICI antitumor activity. However, our clinical cohort included cancers with vastly different expected survival characteristics, potentially obscuring the relationship between specific cytokines and cancer outcomes. Nonetheless, our results support the partial uncoupling of toxicity and benefit and adds to prior evidence identifying IL-6 as a target for irAE interception strategies. These findings require prospective validation in other cohorts and in future clinical trials.

Strengths of the present study include the prospective evaluation of irAEs in a diverse, pan-tumor population. Clinical trials that led the approval of ICIs have generally enrolled a narrow population of patients without baseline autoimmunity (31, 32). The relatively high proportion of patients who are Black and the inclusion of patients with baseline autoimmune disease provides an analysis of the drivers of irAEs in a patient population more representative of clinical practice. The diversity of organs involved, timing, and severity of irAEs combined with the potential for immortal time and survival bias make investigations into irAEs particularly challenging. To address these challenges, we employed time-to-event analyses for irAEs to incorporate timing, which has been rarely performed in other cytokine studies, and multitesting adjustment to investigate the strongest cytokine signals. Further, we also made appropriate adjustments to account for immortal time bias and performed landmark analyses when appropriate, which are common pitfalls in studies investigating irAEs.

There are several limitations to our study. First, we lack the power to make direct conclusions on the granular relationships between distinct cytokine signatures and certain types of organ-specific irAEs. Our subgroup analyses within specific irAE categories are therefore hypothesis generating. Further, grouping irAEs by organ system does not capture the biological diversity of irAE phenotypes within each organ system. Second, while our cohort is diverse, our population has limited numbers of patients with NSCLC in which immunotherapy is a major treatment modality; however, our cohort comprises tumor types such as HCC and RCC, which are featured less prominently in the irAE literature. Third, the cytokine signatures detected in our study likely represent the strongest shared associations with grade ≥ 2 irAEs in a heterogeneously treated cohort, including both dual and single ICI, and do not fully characterize important immunological changes that may precede irAEs and are unique to different treatment regimens, anti-PD-1/PD-L1 based or in combination with anti-CTLA-4 or anti-LAG-3. Fourth, given the heterogeneity of the treatment cohort, survival outcomes may primarily be driven by the cohort tumor type and line of therapy as opposed to immune features, confounding the relationship between cytokines and clinical outcomes. Fifth, there is currently an unmet need to better understand subgroups of patients underrepresented in clinical trials, including patients with prior history of autoimmune diseases and those who identify as Black, and, though our study has higher representation of both groups, we currently do not have the sample size to incorporate these group comparisons along with irAE status into our biomarker analyses. Lastly, due to logistical challenges associated with identifying and promptly obtaining peripheral blood samples from patients with suspected irAEs before extensive steroids or immunosuppression, we had a smaller sample of patients with confirmed irAEs who had available irAE samples for investigation.

In summary, we performed an integrated evaluation of immunological mechanisms and effector cytokines underlying irAEs in a diverse, pan-tumor perspective including tumor types outside of melanoma and NSCLC. These data can inform the design of preclinical and translational studies aimed at further understanding the mechanisms of irAE development while providing the foundation for rationally designed clinical trials evaluating targeted interventions for irAEs.

## Methods

### Sex as a biological variable.

Male and female patients were included in this study; 35.1% of the cohort was female and 64.9% was male. Sex was considered as a covariate in the multi-variate time to event Cox models.

### Study design and clinical definitions.

Full details are provided in the Extended Methods section in the Supplemental Materials.

### Biomarker sample collection, processing, and measurements.

Full details are provided in the Extended Methods section in the Supplemental Materials.

### Data analysis overview.

For evaluation of predictive cytokines at baseline and early on treatment, time-to-event evaluations served as the primary analysis. If there were significant cytokines after mutitesting adjustment,as described in the Statistics section, then follow up secondary analyses were performed. Since patients can develop multiple irAEs concurrently or in succession, the highest grade ≥ 2 irAE for each patient was utilized as the most clinically representative irAE for analysis. Early on-treatment analysis of biomarkers included only month 1 or month 2 samples and excluded patients who developed irAEs prior to their early treatment sample collection.

For analysis of biomarkers important near the time of irAE, the highest grade ≥ 2 irAE for each patient was utilized as the most clinically representative irAE. We selected for samples within ± 4 weeks of irAE onset and ≤ 1 week of steroids or immunosuppression to account for the time it took for irAE detection, study team notification and subsequent sample collection while balancing the confounding effects of immunosuppressive agents on cytokine levels. These irAE samples were compared with the earliest on-treatment sample available for the patients with no grade ≥ 2 irAEs.

### Statistics.

For samples collected on treatment, fold change for each timepoint was calculated relative to baseline to account for interpatient variability. Missing values for cytokine and CyTOF measurements were not inferred and included in analyses. For visualization purposes, log_2_ transformation to concentrations or fold changes of cytokine levels were depicted. Time to event analyses were performed for grade ≥ 2 irAE onset and survival outcomes. In the baseline analysis, time to irAE and survival were defined as time from ICI initiation to event onset. In the early on-treatment analysis, time to irAE and survival were defined as time of blood sample collection to event onset to account for immortal time bias. Patients who were free of the events of interest during the study period were censored at the last follow-up date or death date.

Univariate Cox proportional-hazards models were applied to estimate the HR and corresponding 95% CI for time to event analyses including time to irAE onset and survival. Multivariate Cox analysis was performed to adjust for relevant clinical variables. Cumulative probability of grade ≥ 2 irAEs was estimated via a reverse Kaplan-Meier method, while survival outcomes were estimated with the Kaplan-Meier method. Group differences between Kaplan-Meier curves was tested by using a log-rank test. For the significant cytokine predictors in the primary time-to-event outcome analyses, risk-stratification cut-points (optimal cutoffs) for the corresponding time-to-event outcome was determined with maximally selected log-rank statistics from the time-to-event analysis (39).

For non–time-to-event analyses, to address survivorship bias and enrich for patients with adequate time to develop irAEs, patients were considered not to have developed grade ≥ 2 irAEs only if they had at least 6 months of follow up without evidence of toxicity. We selected this time frame to balance the inclusion of later onset irAEs without limiting our included patients to only exceptional responders. In the cytokine analysis, the Wilcoxon rank-sum test was used when assessing statistical differences between 2 groups, and the Kruskal-Wallis test was used for 3 groups or higher. For the CyTOF analysis, 2 sample *t* test was used to compare means between groups. A Fisher’s exact test was utilized when assessing statistical differences between 2 categorical variables. Box and whisker plots were utilized to show visually the median, interquartile range, minimum, and maximum.

For landmark analyses, grade ≥ 2 irAEs had to have occurred prior to the landmark time, and the outcome event of all-cause death was only considered if it occurred after the landmark time. Associations between grade ≥ 2 irAEs and survival outcomes were assessed via landmark analyses at landmark times of 3 months and 6 months to account for early or late onset grade ≥ 2 irAEs, respectively. For cytokines that were significantly associated with irAE and cancer-specific survival, landmark analyses were performed at 10 weeks for all-cause survival to determine the influence of irAEs on the survival stratification by high and low cytokines. In these analyses, we examined patients who developed the most clinically relevant irAE within the landmark time (10 weeks) after ICI initiation and remained alive after the landmark time. The landmark time of 10 weeks was chosen as the representative time frame for early irAE onset that was after the collection of early treatment samples for the majority of patients.

All statistical tests were 2-sided unless stated otherwise. For the primary statistical analyses that involved multiple comparisons, the Benjamini-Hochberg method was applied, and FDR *P*_adj_ < 0.05 were considered significant (93). Secondary analyses were considered significant at *P* < 0.05. Statistical analyses were performed using RStudio software (Version: 2023.03.0+386).

### Study approval.

The study protocol was approved by the Johns Hopkins Institutional Review Board (IRB #00267960), and all participants provided written informed consent before the blood samples and clinical data were collected.

### Data availability.

Values for all data points, excluding clinical data, in graphs are reported in the Supporting Data Values file. The authors declare that the minimal data set and supporting data files pertaining to clinical data for this study cannot be shared publicly due to ethical and legal restrictions on sharing deidentified data that aligns with the consent of research participants. Current JHU compliance policies require data with no direct consent for public open access sharing be under restricted access. We will provide access through Vivli, an established repository for clinical data that provides open access without a fee restricted to approved researchers under a Data Use Agreement. JHU compliance policy for Vivli requires additional anonymization of certain demographics, including use of age ranges and limiters to outlier values for weight, height, and certain rare diseases, while retaining sufficient value for reference and validation of results. Researchers can request more detailed data from the corresponding author shared though an approved collaboration arrangement.

Aggregated and summary values for key figures and statistical analyses are in tabular form in the Supplemental Materials.

### Code availability.

Only open-source software was used for this study, and no custom code packages were generated for data analysis of cytokine and CyTOF data. Please refer to the Supplemental materials for computing package citations.

## Author contributions

CJK and SC share the first author position for this work due to each individual designing and performing the analyses for cytokines and CyTOF, respectively. CJK was assigned the first position given that CJK also oversaw biospecimen collection and clinical annotation. MY and WJH contributed equally as senior authors to this work. CJK, SLA, M Brancati, HLL, AB, and MN acquired clinical data. KM, KH, SM, JL, and EG performed biospecimen collection, processing, and storage coordination. CT and JG acquired cytokine data. SC, NEG, AH, EMC, SMS, JSB, and GWA acquired the CyTOF data. M Brancati, JD, and BJC were involved in regulatory compliance for the study. HLT performed the biostatistical design of the studies. LD provided bioinformatics support. CJK, EJL, YG, M Baretti, JHC, TYS, and MY recruited and enrolled patients. CJK, SC, MFK, JN, LCC, AP, JB, RGH, AG, SB, LT, EMJ, GSC, RM, WJH, and MY contributed to designing of research studies. CJK and M Brancati managed IRB compliance and clinical database design/administration. CJK designed the cytokine analysis and performed data processing and analysis of cytokine Luminex data. SC and WJH designed the CyTOF studies, and SC analyzed the CyTOF data. GSC, RM, WJH, and WY were involved in the overall design of the study. CJK, MN, and MY adjudicated patient data. The manuscript was written by CJK, SC, WJH, and MY. All authors reviewed, edited, and approved the manuscript.

## Supplementary Material

Supplemental data

ICMJE disclosure forms

Supplemental tables 14-15

Supporting data values

## Figures and Tables

**Table 3:**
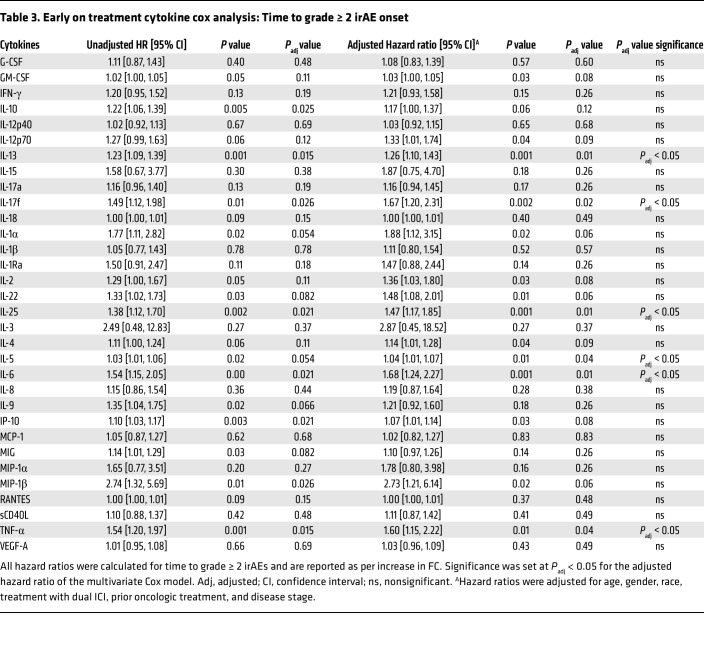
Early on treatment cytokine cox analysis: Time to grade ≥ 2 irAE onset

**Table 2:**
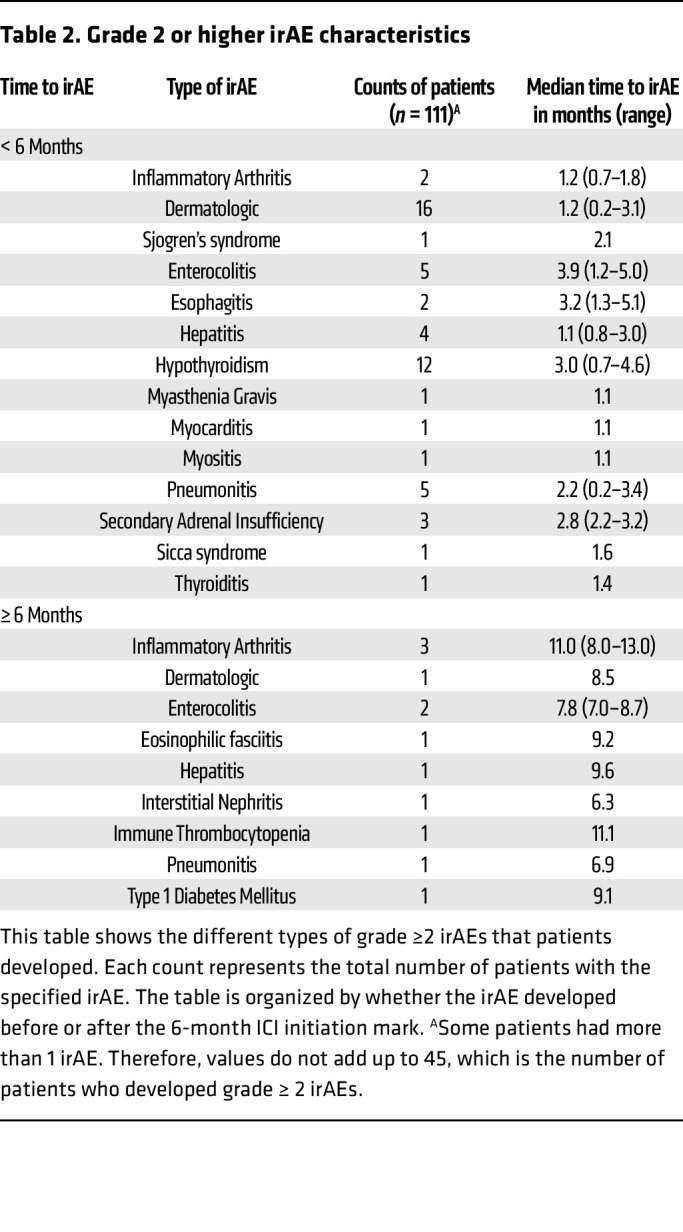
Grade 2 or higher irAE characteristics

**Table 1:**
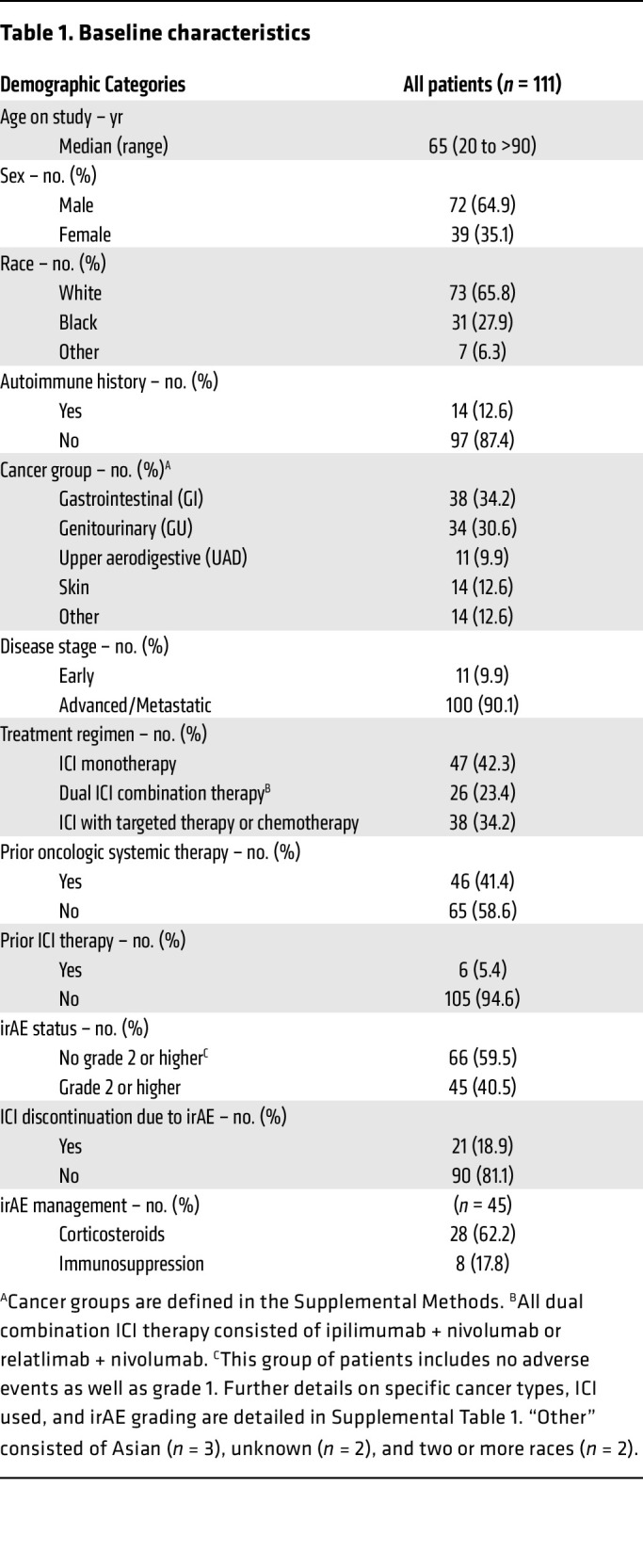
Baseline characteristics

## Version 1 10/15/2024

Electronic publication
