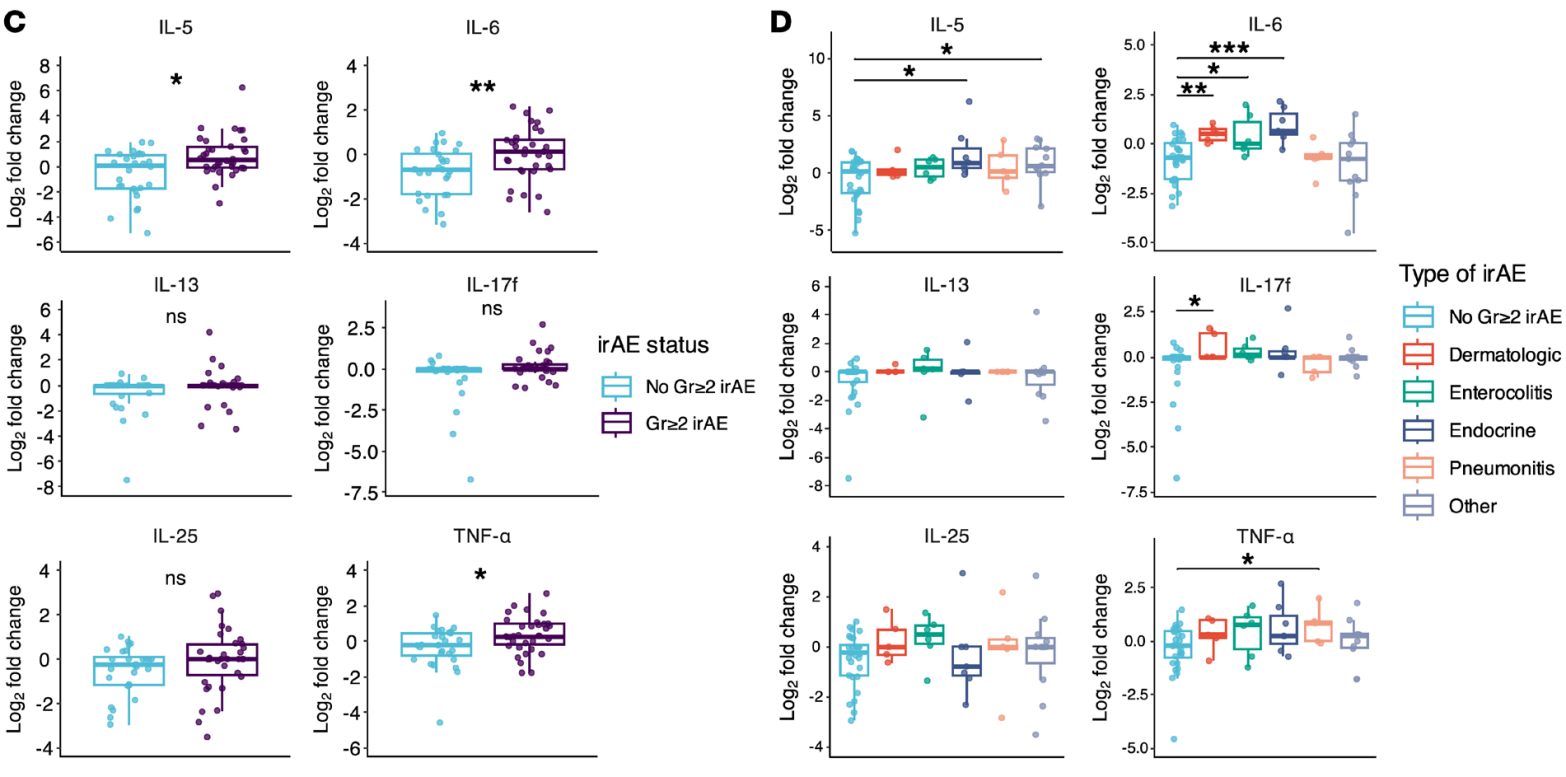# Immune-related events in individuals with solid tumors on immunotherapy associate with Th17 and Th2 signatures

**DOI:** 10.1172/JCI192014

**Published:** 2025-03-17

**Authors:** Chester J. Kao, Soren Charmsaz, Stephanie L. Alden, Madelena Brancati, Howard L. Li, Aanika Balaji, Kabeer Munjal, Kathryn Howe, Sarah Mitchell, James Leatherman, Ervin Griffin, Mari Nakazawa, Hua-Ling Tsai, Ludmila Danilova, Chris Thoburn, Jennifer Gizzi, Nicole E. Gross, Alexei Hernandez, Erin M. Coyne, Sarah M. Shin, Jayalaxmi Suresh Babu, George W. Apostol, Jennifer Durham, Brian J. Christmas, Maximilian F. Konig, Evan J. Lipson, Jarushka Naidoo, Laura C. Cappelli, Aliyah Pabani, Yasser Ged, Marina Baretti, Julie Brahmer, Jean Hoffman-Censits, Tanguy Y. Seiwert, Rachel Garonce-Hediger, Aditi Guha, Sanjay Bansal, Laura Tang, Elizabeth M. Jaffee, G. Scott Chandler, Rajat Mohindra, Won Jin Ho, Mark Yarchoan

Original citation: *J Clin Invest*. 2024;134(20):e176567. https://doi.org/10.1172/JCI176567

Citation for this corrigendum: *J Clin Invest*. 2025;135(6):e192014. https://doi.org/10.1172/JCI192014

The authors recently became aware of errors in the display of box and whisker plots in Figure 4, C and D, Figure 5E, and Figure 7, C and D, and Supplemental Figures 1, 2, and 3, which resulted in data points beyond the tail to appear as double points rather than single points. The authors have stated that none of the underlying data, statistical analyses, results, or conclusions of the article are affected. The authors have provided the correct figure panels below. The HTML and PDF files have been updated to reflect the changes.

The authors regret the errors.

## Figures and Tables

**Figure 7:**
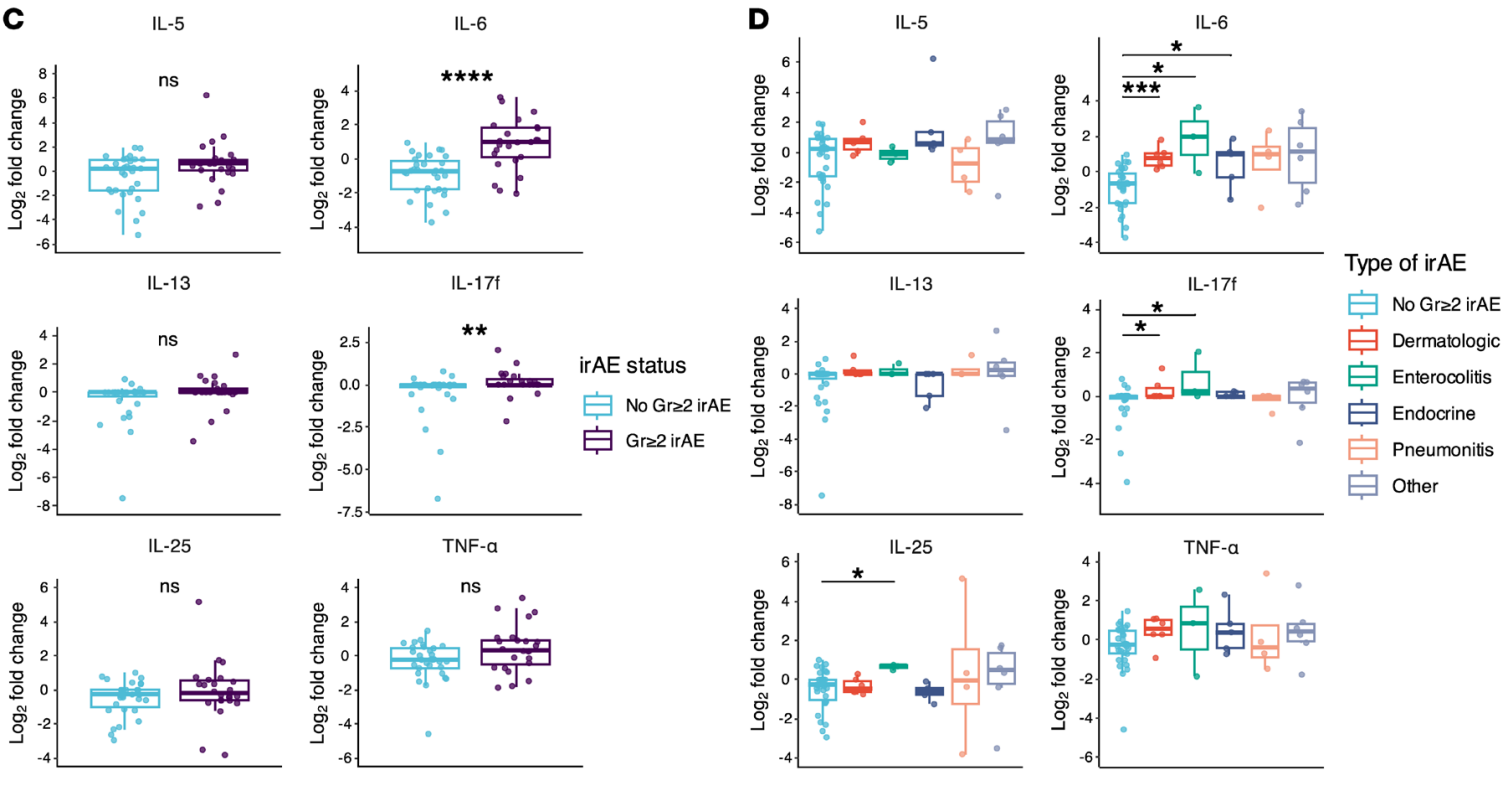


**Figure 5:**
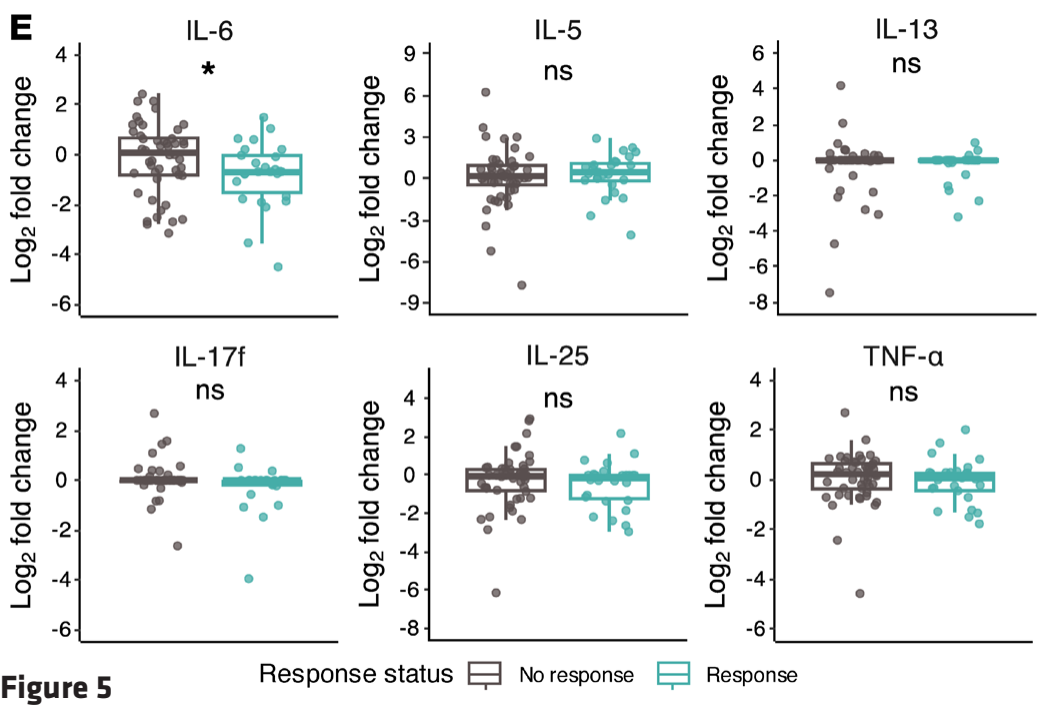


**Figure 4:**