# Supplementary material for: Immune-related events in individuals with solid tumors on immunotherapy associate with Th17 and Th2 signatures
Source: J Clin Invest. 2024 Aug 29;134(20):e176567. doi: 10.1172/JCI176567 (PMC11473156; doi:10.1172/JCI176567)
Supplement: Supplemental data [file jci-134-176567-s223.pdf]

SUPPLEMENTAL MATERIAL

Conflict of interest:

C.J.K is, at the time of the publication, an employee of AstraZeneca. S.S. reports receiving travel honoraria from StandardBiotools. M.F.K was supported by the Rheumatology Research Foundation, the Lupus Research Alliance Lupus Innovation Award, the Harrington Discovery Institute Scholar-Innovator Award, the Stephen and Renee Bisciotti Foundation, the Jerome L. Greene Foundation, the Cupid Foundation, the Sol Goldman MS Research Program, and the National Institute of Allergy and Infectious Diseases/National Institutes of Health (1R21AI176764–01). M.F.K. has received consulting fees from Argenx, Atara Biotherapeutics, Revel Pharmaceuticals, Sana Biotechnology, and Sanofi unrelated to this work. E.J.L reports institutional research funding from Bristol Myers Squibb, Haystack Oncology, Merck, Regeneron, and Sanofi; honoraria/consulting for Bristol Myers Squibb, CareDx, Eisai, Genentech, HUYA Bioscience International, Immunocore, Instil Bio, Lyvgen, Merck, Merck KGaA, Natera, Nektar, Novartis, OncoSec, Pfizer, Rain Therapeutics, Regeneron, Replimune, Sanofi-Aventis, Sun Pharma, Syneos Health; and stock ownership in Iovance. J.N. reports research funding from AstraZeneca, Bristol Myers Squibb, Roche/Genentech, Amgen, Arcus Biosciences, Pfizer, Takeda and Mirati; honoraria from AstraZeneca, Bristol Myers Squibb, Daiichi Sankyo, Roche/Genentech, and Mirati; and serving on consulting/advisory boards for the previously described relationships along with NGM Pharmaceuticals, Bayer, Regeneron, Takeda, Pfizer, Elevation Oncology, Abbvie, and Kaleido Biosciences. A.P. reports grants and personal fees from Bristol-Myers Squibb; personal fees from AstraZeneca, Pfizer, Merck, Roche, and Canadian Agency for Drugs and Technologies in Health; and grants from Alberta Cancer Foundation outside the submitted work. M.Ba. reports consulting fees from AstraZeneca. J.B reports institutional research funding from AstraZeneca. E.M.J. reports other support from Abmeta, other support from Adventris, personal fees from Achilles, DragonFly, Neuvogen, Parker Institute, CPRIT, Surge, Mestag, Medical Home Group, and HDTbio, grants from Lustgarten, grants from Genentech, BMS, NeoTX, and Break Through Cancer outside the submitted work. E.M.J. is the Dana and Albert "Cubby" Broccoli Professor of Oncology. R.G.H, G.S.C., R.M. are employees of F. Hoffman-La Roche Ltd., and A.G., S.B, and L.T. are employees of Genentech Inc. W.J.H. has received patent royalties from Rodeo/ Amgen and grants from Sanofi, NeoTX, and CirclePharma. W.J.H has received speaking/travel honoraria from Exelixis and Standard BioTools. M.Y reports research funding (to Johns Hopkins) from Genentech, Bristol-Myers Squibb, and Incyte; consulting fees from AstraZeneca, Exelixis, Genentech, Replimune, Hepion, Lantheus; and is a co-founder with equity of Adventris Pharmaceuticals. The other authors declare no competing interests.

SUPPLEMENTAL MATERIAL

Table of Contents

EXTENDED METHODS 3
Additional Clinical definitions 3
Racial and Ethnic Categories 3
Study design 5
Clinical definitions 5
Plasma Sample Collection 6
Cytokine Measurements 6
Antibodies 6
CyTOF Staining, Acquisition, and Analyses 6
Table S1: Expanded baseline characteristics in the total cohort 8
Table S2: Details of baseline autoimmune history 9
Figure S1. Baseline cytokine differences between patients based on baseline autoimmune history 10
Figure S2. Baseline cytokine differences based on prior exposure to oncologic systemic therapy 11
Figure S3. Baseline cytokine differences based on cancer group 12
Table S3. Baseline cytokine cox analysis: Time to grade ≥2 irAE onset 13
Table S4. Characteristics of the early on treatment cytokine cohort: Time to event analysis. 14
Table S5. Early on treatment cytokine cox analysis: Time to grade ≥2 irAE onset for dual ICI cohort 15
Table S6. Early on treatment cytokine cox analysis: Time to grade ≥2 irAE onset for single ICI cohort 16
Table S7. Characteristics of the early on treatment cytokine cohort: Wilcoxon analysis. 17
Table S8. Wilcoxon analysis: early on treatment cytokine and irAE 18
Table S9. Wilcoxon analysis: early on treatment cytokine and response 19
Table S10. Early on treatment cytokine cox analysis: cancer-specific survival 20
Table S11. Early on treatment cytokine cox analysis: overall survival 21
Table S12. Characteristics of the time of irAE cytokine cohort: Wilcoxon analysis. 22

Table S13. Wilcoxon analysis: time of irAE cytokines 23
Table S14. CyTOF antibody panel 24
Table S15. CyTOF clustering annotations 24
Table S16. Characteristics of the overall CyTOF cohort. 25
Figure S4. Diagnostic plots indicating success of CyTOF sample normalization. 26
Table S17. CyTOF analysis: baseline 27
Table S18. CyTOF analysis: on treatment 28
Table S19. CyTOF analysis: fold change 29
References 30
Key Resources 31

EXTENDED METHODS

Inclusion and exclusion criteria

Inclusion criteria
- Subject has pathologically confirmed or clinically suspected neoplasm
- Subject is receiving immune checkpoint therapy as standard of care
- Age ≥ 18 years old
- Ability to understand and willingness to sign a written informed consent document

Exclusion criteria
- Subject has contraindications for venipuncture (e.g. subjects who are clinically unstable, subjects with hemoglobin < 7.0 g/dL). Subjects who do not meet this criterion may still participate in this study but will be excluded from the blood collection component.
- Total volume of blood donation on all studies (including the present study) exceeds a safe parameter (220 CCs within a month). Subjects who do not meet this criterion may still participate in this study but will be excluded from the blood collection component.

Additional Clinical definitions

Racial and Ethnic Categories

Self-reported race information was extracted from the electronic medical record (EMR). Options included White, Black, Asian, Native Hawaiian or Other Pacific Islander, American Indian or Alaska Native, two or more races, not reported, other, or unknown. For the purposes of multivariate statistical analyses, we grouped race into three categories: White, Black, and Other. The Other group consisted of Asian, Native Hawaiian or Other Pacific Islander, American Indian or Alaska Native, two or more races, not reported, other, or unknown.

Cancer group definitions

**Gastrointestinal**	**Genitourinary**	**Upper aerodigestive**	**Skin**	**Other**
Biliary tract	Bladder	Head and neck	Cutaneous squamous cell	Adrenal
Pancreatic	Prostate	Lung	Melanoma	Breast
Hepatocellular	Renal		Merkel cell	Cervical
Colorectal				Endometrial
Gastric				Sarcoma
Gastrointestinal neuroendocrine				Vulvovaginal
Esophagogastric				

Grouped specific irAEs

Given the limited numbers of certain types of irAEs, we grouped irAEs within the best matched organ system. For the purposes of the exploratory group comparisons, we only included grouped organ-specific irAEs in those analyses if the count was 3 or higher; otherwise, irAEs were grouped within the other category.

**Dermatologic**	**Enterocolitis**	**Endocrine**	**Pneumonitis**	**Other**
Alopecia	Colitis	Hypothyroidism	Pneumonitis	Arthritic
Bullous pemphigoid		Secondary adrenal insufficiency		Eosinophilic fasciitis
Lichenoid dermatitis		Thyroiditis		Hepatitis
Maculopapular eruption		Type 1 diabetes mellitus		Interstitial nephritis
Rash, NOS				Immune thrombocytopenia
Urticarial eruption				Myasthenia gravis
				Myocarditis
				Sicca syndrome
				Sjogren's syndrome

Abbreviation: NOS = not otherwise specified.

Study design

We are conducting an ongoing prospective observational study of patients with solid tumors who received immune checkpoint inhibitor (ICI) treatment as standard of care at Johns Hopkins University from May 2021 to the present. Eligible patients were aged > 18 years with pathologically confirmed solid tumors treated with ICI consisting of anti-PD-1/PD-L1 blockade (nivolumab, pembrolizumab, atezolizumab, cemiplimab, durvalumab, and avelumab), combination ICI blockade with anti-PD-1 (nivolumab) and anti-CTLA-4 (ipilimumab) or anti-PD-1 (nivolumab) and anti-LAG-3 (relatlimab), or in combination with targeted therapy or chemotherapy. All patients enrolled in the study had peripheral blood samples collected at baseline prior to initiation of the ICI. Subsequently, peripheral blood samples were collected at month 1, 2, 4, 6, and 12 as long as the patient was continued on ICI and if available. Peripheral blood was also collected at each irAE event if possible. Patients were clinically followed up to one year after their last dose of ICI to ensure adequate follow up for late onset irAEs.

Clinical definitions

Patient characteristics were abstracted from the electronic medical record (EMR) including age, sex, race/ethnicity, cancer histology, baseline autoimmune history, prior oncologic treatment history, treatment setting (advanced/metastatic vs neoadjuvant/adjuvant), and type and dates of ICI treatments. Cancer groups for GI, GU, UAD, skin, and other are defined in the Extended methods section of the Supplemental. IrAEs were defined based on Common Terminology Criteria for Adverse Events version 5 (CTCAE v.5.0). The dates of onset, grade, and duration of irAEs were determined from review by a clinical researcher and confirmed by a medical oncologist reviewer. For group comparisons of organ-specific irAEs, we separated irAEs into dermatologic, enterocolitis, endocrine, pneumonitis, and other irAEs (Supplemental: Extended methods). Since grade 1 irAEs are mild and generally asymptomatic, and intervention is usually not indicated; we focused on grade ≥2 irAEs as the main endpoint to enrich for clinically relevant irAEs and reduce confounding for patients who received ICI in combination with other therapies. Only grade ≥ 2 irAEs were utilized in the analyses, and patients with no grade ≥2 irAEs included both no irAEs and grade 1 irAEs. Best responses were based on RECIST v1.1 criteria and treating oncologists' documentation. Responses were defined as partial or complete response, while non-responses were defined as stable disease or progression. The best response was the nadir achieved when comparing on-treatment imaging to the baseline imaging prior to ICI initiation. The frequency of imaging obtained was per standard of care and at the discretion of the treating oncologist. Survival outcomes of interest included cancer-specific and all-cause death.

Plasma Sample Collection

Blood samples were obtained in heparinized syringes by standard phlebotomy technique and processed within 2 hours of collection. For the isolation of plasma, blood was transferred into a 50-ml conical tube and placed in a centrifuge at 2,000 r.p.m. for 20 min with the brakes off. The plasma layer was removed and stored in 1-ml aliquots at −80 °C. Peripheral blood mononuclear cells (PBMCs) from the remaining blood were isolated using a standard LeucoSep tube technique. Briefly, blood diluted in equal parts of PBS was added to LeucoSep tubes preloaded with Ficoll-Paque. The tubes were then centrifuged for 20 min at 2,000 r.p.m. with no brake. The PBMC suspension was collected, the cells were washed in PBS and stored in a cryovial initially at −80 °C before transfer to liquid nitrogen for long-term storage.

Cytokine Measurements

The Bioplex 200 platform (Biorad, Hercules CA) was used to determine the concentration (pg/mL) of multiple target cytokines in plasma. Luminex bead-based immunoassays (Millipore, Billerica NY) were performed following the Johns Hopkins Immune Monitoring Core SOPs and concentrations were determined using 5 parameter log curve fits (using Bioplex Manager 6.0) with vendor-provided standards and quality controls. The HCYTA-60K panel was used to detect 32 cytokines (sCD40L, IL-1α, IL-1β, IL-1Ra, IL-2, IL-3, IL-4, IL-5, IL-6, IL-8, IL-9, IL-10, IL-12p40, IL-12p70, IL-13, IL-15, IL-17a, IL-17f, IL-18, IL-22, IL-25, G-CSF, GM-CSF, TNF-α, IFN-γ, MCP-1, MIP-1α, MIP-1β, RANTES, MIG, IP-10, VEGF-A). Concentrations which were outside of the standard curves values were categorized as “out of range” (OOR). For each cytokine, OOR< values were replaced with the lower limit of the standard curve of the assay while OOR> were replaced with the upper limit of the standard curve. To account for batch effect and ensure accurate fold change calculations, on-treatment samples of each patient were always run with the corresponding baseline sample. For patients with baseline samples that were run in multiple batches, the average concentration of those baseline samples was used in baseline analyses.

Antibodies

All antibodies used for CyTOF are listed in Supplemental Table 14. Custom antibodies were conjugated as previously described (94).

CyTOF Staining, Acquisition, and Analyses

Patient PBMCs were thawed in a 37°C water bath and gradually recovered using pre-warmed RPMI containing 10% FBS. Samples were then counted and $2x10^6$ cells from each sample were plated in 96-well plates. Cells were allowed to rest in the media for 30 minutes prior to staining. Cells were then washed once in PBS with 2mM EDTA. Next, cells were incubated for 2.5 minutes at room temperature in 20uM Pt (Standard BioTools) in PBS to mark for viability. Following the 2.5 minutes, RPMI containing 10% FBS was added to the cells to quench any residual platinum. This is followed by two washes with

cell staining buffer (CSB) (Standard BioTools). Following washes, all samples were barcoded by incubating the cells with unique combinations of metal conjugated anti-CD45 antibodies for 20 minutes. After 2 washes with CSB, samples were multiplexed and transferred to v-bottom flow tubes using a 40um filter. Each tube is then blocked using anti-human FcR block (12ul used for $15x10^6$ cells) for 10 minutes at room temperature. This was followed by a chemokine stain cocktail (Supplemental Table 14) for 10 minutes in a 37°C water bath. Tubes were removed from the water bath and a surface stain cocktail (Supplemental Table 14) was added for 30 minutes at room temperature. Samples were washed twice with CSB and then fixed and permeabilized using cytofix/cytoperm solution (BD Biosciences) for 30 minutes at room temperature. Fixed and permeabilized samples were then washed with perm/wash solution (BD Biosciences) and then subsequently stained using the intracellular cocktail (Supplemental Table 14) for 30 minutes at room temperature. Samples were washed twice with perm/wash solution and stored in 1.6% PFA in PBS at 4°C until the day of acquisition, not exceeding one week. On the day of acquisition, samples were stained with 1:500 ^{103}Rh in Maxpar Fix/Perm solution (Standard BioTools) for 30 minutes at room temperature for cell identification. Samples were washed with PBS once and then washed and resuspended in normalization beads (Standard BioTools). The data were acquired on a Helios mass cytometer (Standard BioTools) at the Johns Hopkins University Mass Cytometry Facility. All acquired data was randomized and normalized using CyTOF software (v7.1.16389.0, Standard BioTools). Resulting fcs files were then debarcoded by manual gating using FlowJo software (v10.9.0, BD Biosciences). Cell events were gated using ^{103}Rh positivity. Live cells were then gated based on ^{194}Pt and ^{195}Pt viability staining. This was followed by debarcoding based on positivity of unique combinations of CD45 barcodes. Each debarcoded sample was then exported as an individual fcs file. Samples were normalized in R (v4.0.2) using the CytoNorm algorithm that utilized a repeated sample included in each staining batch to normalize all samples based on goal quantiles of mean marker expression (95). Clustering analysis was performed in R using FlowSOM to generate 40 metaclusters that were annotated using the expression profile of markers included within the panel, resulting in 27 final clusters (96).

Table S1: Expanded baseline characteristics in the total cohort

Characteristics	Total patients (n = 111)
**Cancer type - no. (%)**	
Adrenal	1 (0.9)
Biliary Tract	2 (1.8)
Bladder	10 (9.0)
Breast	3 (2.7)
Cervical	2 (1.8)
Colorectal	1 (0.9)
Endometrial	2 (1.8)
Esophagogastric Junction	1 (0.9)
Head and Neck	9 (8.1)
Hepatocellular Carcinoma	30 (27.0)
Lung	2 (1.8)
Melanoma	9 (8.1)
Neuroendocrine	3 (2.7)
Pancreas	1 (0.9)
Renal Cell Carcinoma	24 (21.6)
Sarcoma	5 (4.5)
Squamous Cell Carcinoma of Skin	5 (4.5)
Vulvovaginal	1 (0.9)
**irAE grade - no. (%)**	
No grade 2 or higher[A]	66 (59.5)
Grade 2	22 (19.8)
Grade 3	16 (14.4)
Grade 4	5 (4.5)
Grade 5	2 (1.8)
**ICI treatment - no. (%)**	
Anti-PD-1 or anti-PD-L1	
Atezolizumab	17 (15.3)
Cemiplimab	5 (4.5)
Nivolumab	15 (13.5)
Pembrolizumab	48 (43.2)
Anti-CTLA-4 and anti-PD-1	
Ipilimumab + Nivolumab	24 (21.6)
Anti-LAG-3 and anti-PD-1	
Relatlimab + Nivolumab	2 (1.9)

[A]This group of patients includes no adverse events as well as grade 1.

Table S2: Details of baseline autoimmune history

Characteristics	Total patients (*n* = 14)
**Type of autoimmune disorder**, ***n*** [A]	
Crohn's disease	2
Episcleritis/scleritis	1
Graves' disease	2
Hashimoto's thyroiditis	2
Hypertrophic lichen planus	1
IgA mediated leukocytoclastic vasculitis	1
Psoriasis	3
Rheumatoid arthritis	3
Sjogren's syndrome	1
**On steroids/DMARDs for the autoimmune disorder at time of consent – no. (%)**	
Yes	4 (28.6%)
No	10 (71.4%)

[A]Two patients had two concurrent autoimmune disorders, so the cases of autoimmune disorders at baseline do not equal n=14. One patient had psoriasis along with rheumatoid arthritis, and the other patient had episcleritis/scleritis with Hashimoto's thyroiditis. Abbreviations: DMARDs = disease-modifying anti-rheumatic drugs

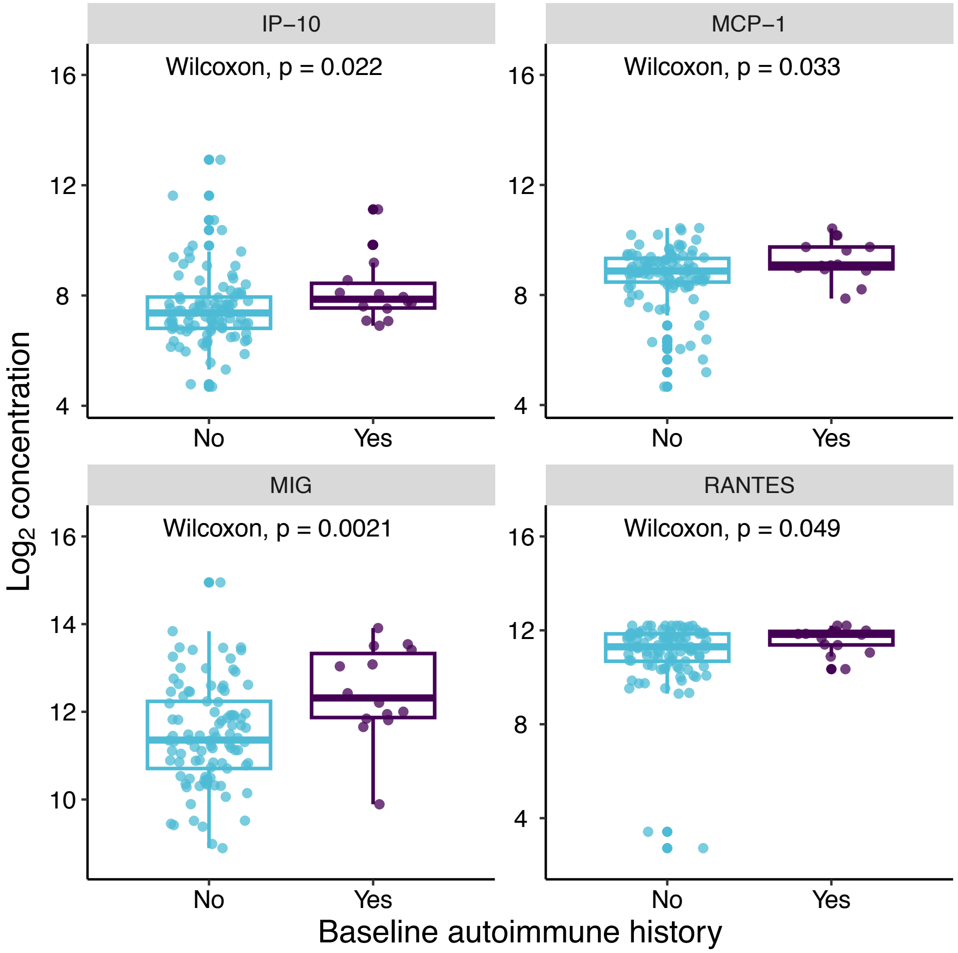

Figure S1. Baseline cytokine differences between patients based on baseline autoimmune history Plotted are $\log_2$ transformed baseline concentrations, measured in pg/mL, of significant cytokines based on Wilcoxon rank-sum test.

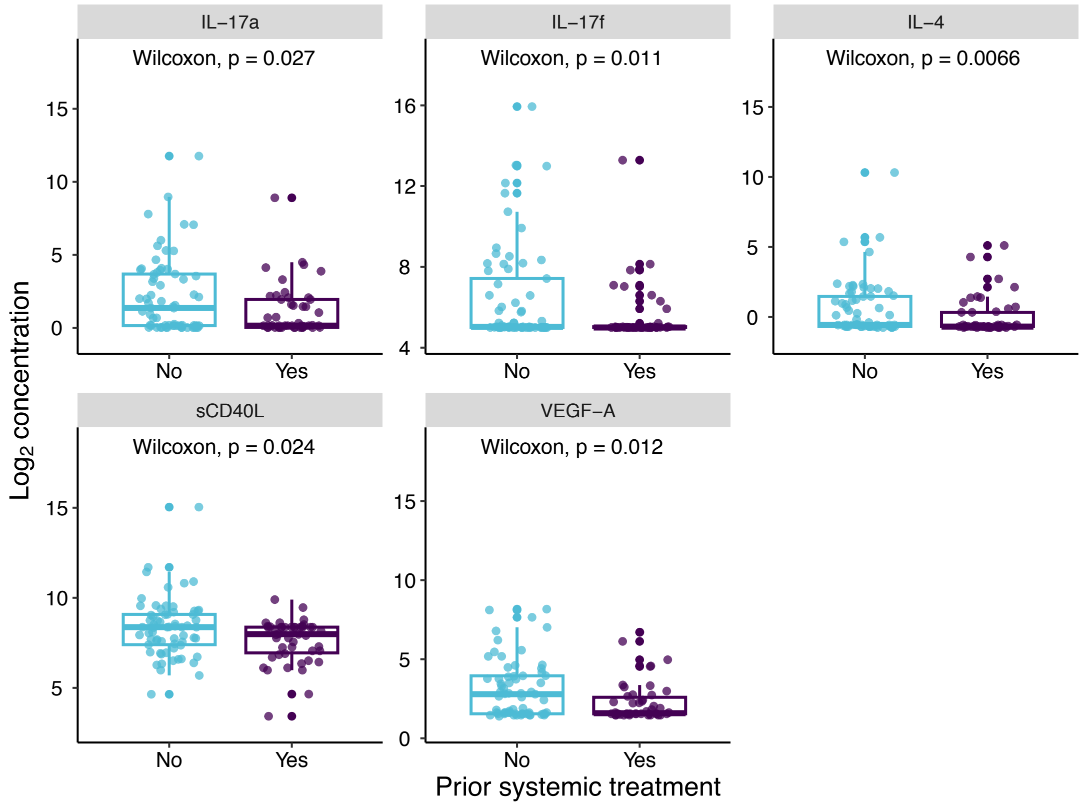

Figure S2. Baseline cytokine differences based on prior exposure to oncologic systemic therapy
Plotted are log_2 transformed baseline concentrations, measured in pg/mL, of significant cytokines based on Wilcoxon rank-sum test.

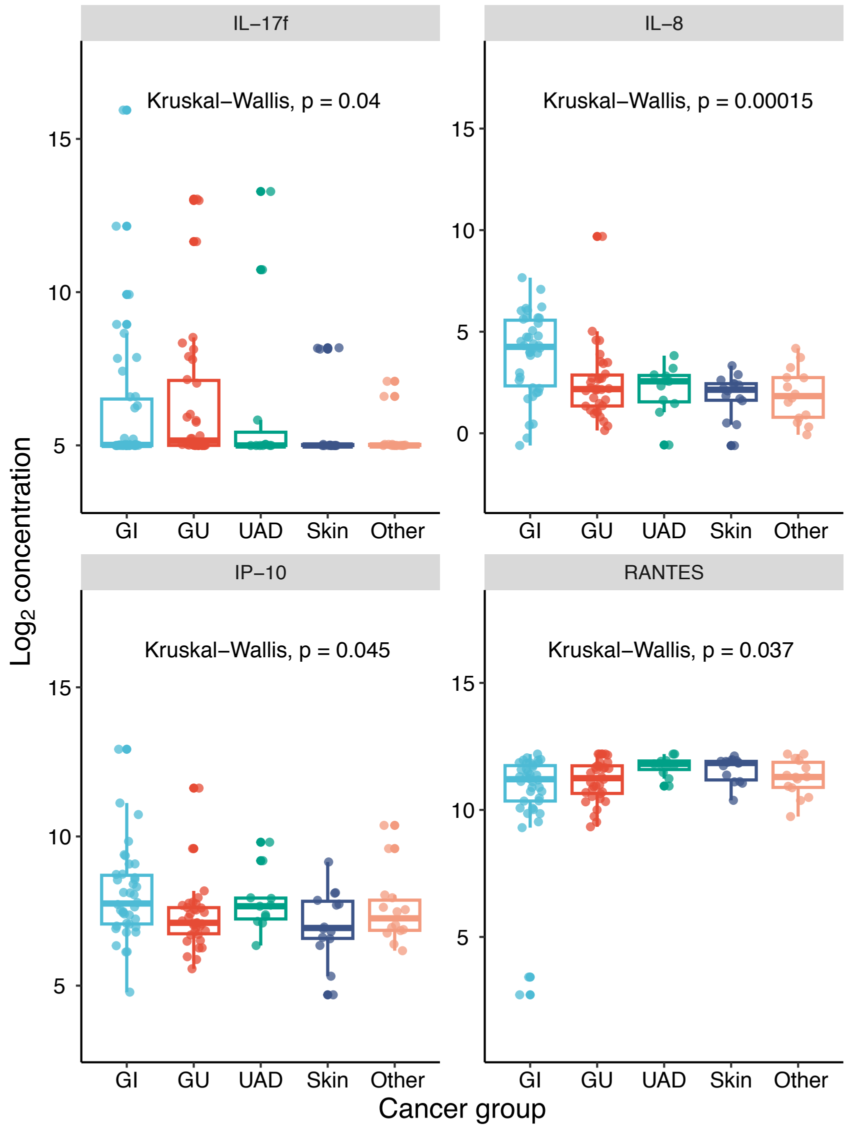

Figure S3. Baseline cytokine differences based on cancer group
Plotted are $\log_2$ transformed baseline concentrations, measured in pg/mL, of significant cytokines. Kruskal-Wallis test was used to compare all groups. Abbreviations: GI = gastrointestinal, GU = genitourinary, UAD = upper aerodigestive.

Table S3. Baseline cytokine cox analysis: Time to grade ≥2 irAE onset

Cytokines	Unadjusted Hazard ratio [95% CI]	P-value	Adjusted P-value	Adjusted Hazard ratio [95% CI][A]	P-value	Adjusted P-value	Adj. P-value significance
G-CSF	1.00 [0.99, 1.00]	0.34	0.74	1.00 [0.99, 1.00]	0.38	0.75	ns
GM-CSF	1.00 [1.00, 1.00]	0.48	0.74	1.00 [1.00, 1.00]	0.47	0.75	ns
IFN-γ	1.00 [1.00, 1.00]	0.43	0.74	1.00 [1.00, 1.00]	0.35	0.75	ns
IL-10	1.00 [0.99, 1.00]	0.46	0.74	1.00 [0.99, 1.00]	0.49	0.75	ns
IL-12p40	1.00 [1.00, 1.00]	0.66	0.74	1.00 [1.00, 1.00]	0.77	0.80	ns
IL-12p70	1.00 [0.99, 1.00]	0.46	0.74	1.00 [0.99, 1.00]	0.53	0.75	ns
IL-13	1.00 [1.00, 1.00]	0.40	0.74	1.00 [1.00, 1.00]	0.37	0.75	ns
IL-15	1.00 [0.99, 1.01]	0.59	0.74	1.00 [0.99, 1.01]	0.61	0.75	ns
IL-17a	1.00 [1.00, 1.00]	0.58	0.74	1.00 [1.00, 1.00]	0.64	0.75	ns
IL-17f	1.00 [1.00, 1.00]	0.44	0.74	1.00 [1.00, 1.00]	0.49	0.75	ns
IL-18	1.00 [1.00, 1.00]	0.67	0.74	1.00 [1.00, 1.00]	0.48	0.75	ns
IL-1α	1.00 [1.00, 1.00]	0.57	0.74	1.00 [1.00, 1.00]	0.62	0.75	ns
IL-1β	1.00 [0.99, 1.00]	0.39	0.74	1.00 [0.99, 1.00]	0.45	0.75	ns
IL-1Ra	0.99 [0.99, 1.00]	0.30	0.74	1.00 [0.99, 1.00]	0.36	0.75	ns
IL-2	0.99 [0.97, 1.02]	0.54	0.74	0.99 [0.97, 1.02]	0.62	0.75	ns
IL-22	1.00 [1.00, 1.00]	0.54	0.74	1.00 [1.00, 1.00]	0.58	0.75	ns
IL-25	1.00 [1.00, 1.00]	0.46	0.74	1.00 [1.00, 1.00]	0.52	0.75	ns
IL-3	0.71 [0.43, 1.19]	0.19	0.74	0.71 [0.41, 1.22]	0.21	0.75	ns
IL-4	0.95 [0.88, 1.03]	0.25	0.74	0.96 [0.88, 1.04]	0.28	0.75	ns
IL-5	1.00 [0.99, 1.01]	0.60	0.74	1.00 [0.99, 1.01]	0.68	0.75	ns
IL-6	1.00 [0.99, 1.01]	0.73	0.78	1.00 [0.99, 1.01]	0.82	0.82	ns
IL-8	1.00 [0.99, 1.00]	0.53	0.74	1.00 [0.99, 1.00]	0.60	0.75	ns
IL-9	1.00 [0.99, 1.01]	0.83	0.85	1.00 [0.99, 1.01]	0.66	0.75	ns
IP-10	1.00 [1.00, 1.00]	0.66	0.74	1.00 [1.00, 1.00]	0.51	0.75	ns
MCP-1	1.00 [1.00, 1.00]	0.27	0.74	1.00 [1.00, 1.00]	0.16	0.75	ns
MIG	1.00 [1.00, 1.00]	0.85	0.85	1.00 [1.00, 1.00]	0.70	0.75	ns
MIP-1α	1.00 [0.99, 1.00]	0.28	0.74	1.00 [0.99, 1.00]	0.26	0.75	ns
MIP-1β	0.98 [0.96, 1.00]	0.03	0.74	0.98 [0.96, 1.00]	0.04	0.75	ns
RANTES	1.00 [1.00, 1.00]	0.66	0.74	1.00 [1.00, 1.00]	0.33	0.75	ns
sCD40L	1.00 [1.00, 1.00]	0.55	0.74	1.00 [1.00, 1.00]	0.59	0.75	ns
TNF-α	1.00 [0.99, 1.00]	0.28	0.74	1.00 [0.99, 1.00]	0.33	0.75	ns
VEGF-A	0.99 [0.98, 1.00]	0.15	0.74	0.99 [0.98, 1.00]	0.21	0.75	ns

All hazard ratios are reported as per increase in pg/ml. Significance was set at adjusted $P < 0.05$. Abbreviations: Adj. = adjusted, CI = confidence interval, ns = non-significant.
[A]Hazard ratios were adjusted for baseline autoimmune history and prior systemic therapy.

Table S4. Characteristics of the early on treatment cytokine cohort: Time to event analysis.

Characteristics	Total patients (n = 88)
**Age on study (years)**	
Median (range)	66 (20 to >90)
**Sex - no. (%)**	
Female	29 (33.0)
Male	59 (67.0)
**Race - no. (%)**	
White	59 (67.0)
Black	24 (27.3)
Other	5 (5.7)
**Autoimmune history - no. (%)**	
Yes	10 (11.4)
No	78 (88.6)
**Cancer type - no. (%)**	
Adrenal	1 (1.1)
Biliary Tract	2 (2.3)
Bladder	8 (9.1)
Breast	2 (2.3)
Cervical	2 (2.3)
Colorectal	1 (1.1)
Endometrial	1 (1.1)
Esophagogastric Junction	1 (1.1)
Head and Neck	8 (9.1)
Hepatocellular Carcinoma	23 (26.1)
Lung	2 (2.3)
Melanoma	6 (6.8)
Neuroendocrine	2 (2.3)
Pancreas	0 (0.0)
Renal Cell Carcinoma	20 (22.7)
Sarcoma	3 (3.4)
Squamous Cell Carcinoma of Skin	5 (5.7)
Vulvovaginal	1 (1.1)
**Disease stage - no. (%)**	
Early	9 (10.2)
Advanced/Metastatic	79 (89.8)
**Immune checkpoint inhibitor - no. (%)**	
anti-PD-1 or anti-PD-L1	
Atezolizumab	14 (15.9)
Cemiplimab	5 (5.7)
Nivolumab	13 (14.7)
Pembrolizumab	38 (43.2)
anti-CTLA-4 and anti-PD-1	
Ipilimumab + Nivolumab	17 (19.3)
anti-LAG-3 and anti-PD-1	
Relatlimab + Nivolumab	1 (1.2)
**Treatment regimen - no. (%)**	
ICI Monotherapy	36 (40.9)
ICI Combination Therapy	18 (20.5)
ICI with Targeted Therapy or Chemotherapy	34 (38.6)
**Prior systemic therapy - no. (%)**	
Yes	34 (38.6)
No	54 (61.4)
**Prior ICI therapy - no. (%)**	
Yes	4 (4.5)
No	84 (95.5)
**Max irAE status - no. (%)**	
Grade 2 or higher irAE	34 (38.6)
No grade 2 or higher irAE	54 (61.4)

Table S5. Early on treatment cytokine cox analysis: Time to grade ≥2 irAE onset for dual ICI cohort

Cytokines	Unadjusted Hazard ratio [95% CI]	P-value	Adjusted P-value	Adjusted Hazard ratio [95% CI][A]	P-value	Adjusted P-value	Adj. P-value significance
G-CSF	0.98 [0.63, 1.54]	0.94	0.94	1.14 [0.70, 1.86]	0.60	0.74	ns
GM-CSF	1.55 [0.77, 3.10]	0.22	0.74	1.82 [0.81, 4.13]	0.15	0.42	ns
IFN-γ	1.15 [0.61, 2.16]	0.67	0.76	1.96 [0.81, 4.75]	0.13	0.42	ns
IL-10	1.14 [0.93, 1.40]	0.21	0.74	1.00 [0.77, 1.30]	0.98	0.98	ns
IL-12p40	1.18 [0.87, 1.59]	0.28	0.74	1.30 [0.89, 1.92]	0.18	0.43	ns
IL-12p70	0.53 [0.07, 3.82]	0.53	0.74	0.91 [0.02, 40.86]	0.96	0.98	ns
IL-13	0.26 [0.03, 2.20]	0.22	0.74	0.36 [0.01, 11.29]	0.56	0.74	ns
IL-15	0.11 [0.01, 2.15]	0.15	0.74	3.19 [0.01, 1673.72]	0.72	0.82	ns
IL-17a	1.10 [0.68, 1.76]	0.71	0.76	1.34 [0.78, 2.32]	0.29	0.47	ns
IL-17f	3.43 [0.16, 74.37]	0.43	0.74	48.29 [0.29, 7945.97]	0.14	0.42	ns
IL-18	1.00 [1.00, 1.01]	0.41	0.74	1.00 [1.00, 1.01]	0.29	0.47	ns
IL-1α	0.46 [0.07, 2.90]	0.41	0.74	1.48 [0.09, 23.44]	0.78	0.86	ns
IL-1β	1.09 [0.20, 5.91]	0.92	0.94	9.58 [0.43, 214.50]	0.15	0.42	ns
IL-1Ra	1.95 [0.89, 4.24]	0.09	0.74	4.82 [0.84, 27.69]	0.08	0.42	ns
IL-2	0.64 [0.06, 6.72]	0.71	0.76	3.14 [0.03, 298.97]	0.62	0.74	ns
IL-22	1.46 [0.33, 6.42]	0.61	0.76	2.20 [0.53, 9.16]	0.28	0.47	ns
IL-25	1.36 [0.83, 2.24]	0.23	0.74	1.97 [0.90, 4.32]	0.09	0.42	ns
IL-3[B]							
IL-4	2.07 [0.18, 23.38]	0.56	0.75	14.49 [0.34, 626.67]	0.16	0.42	ns
IL-5	1.16 [0.88, 1.53]	0.29	0.74	1.22 [0.86, 1.71]	0.26	0.47	ns
IL-6	1.46 [0.65, 3.31]	0.36	0.74	1.65 [0.61, 4.44]	0.32	0.48	ns
IL-8	1.40 [0.52, 3.73]	0.50	0.74	1.88 [0.51, 6.97]	0.35	0.49	ns
IL-9	1.11 [0.80, 1.55]	0.53	0.74	1.29 [0.82, 2.01]	0.27	0.47	ns
IP-10	1.07 [0.99, 1.16]	0.08	0.74	1.22 [1.00, 1.48]	0.05	0.42	ns
MCP-1	0.83 [0.52, 1.32]	0.43	0.74	0.99 [0.39, 2.53]	0.98	0.98	ns
MIG	1.12 [0.96, 1.31]	0.15	0.74	1.48 [1.07, 2.04]	0.02	0.42	ns
MIP-1α	1.71 [0.19, 15.75]	0.64	0.76	196.43 [1.52, 25458.96]	0.03	0.42	ns
MIP-1β	2.14 [0.37, 12.46]	0.40	0.74	7.32 [0.68, 78.87]	0.10	0.42	ns
RANTES	1.00 [1.00, 1.01]	0.41	0.74	1.00 [1.00, 1.01]	0.29	0.47	ns
sCD40L	0.85 [0.42, 1.73]	0.65	0.76	0.78 [0.32, 1.90]	0.58	0.74	ns
TNF-α	1.24 [0.65, 2.38]	0.51	0.74	4.07 [0.88, 18.74]	0.07	0.42	ns
VEGF-A	1.55 [0.44, 5.51]	0.50	0.74	3.08 [0.36, 26.34]	0.30	0.47	ns

All hazard ratios are reported as per increase in fold change. Significance was set at adjusted $P < 0.05$. Abbreviations: Adj. = adjusted, CI = confidence interval, ICI = immune checkpoint inhibitor, ns = non-significant.
[A]Hazard ratios were adjusted for age, gender, race, prior oncologic treatment, and disease stage. [B]Hazard ratios for IL-3 could not be calculated due to insufficient data.

Table S6. Early on treatment cytokine cox analysis: Time to grade ≥2 irAE onset for single ICI cohort

Cytokines	Unadjusted Hazard ratio [95% CI]	P-value	Adjusted P-value	Adjusted Hazard ratio [95% CI][A]	P-value	Adjusted P-value	Adj. P-value significance
G-CSF	1.20 [0.90, 1.60]	0.22	0.28	1.22 [0.90, 1.66]	0.19	0.26	ns
GM-CSF	1.03 [1.01, 1.05]	0.02	0.04	1.03 [1.00, 1.06]	0.03	0.05	ns
IFN-γ	1.26 [0.97, 1.64]	0.08	0.13	1.31 [0.97, 1.76]	0.07	0.12	ns
IL-10	1.21 [0.98, 1.50]	0.07	0.12	1.26 [1.00, 1.58]	0.05	0.09	ns
IL-12p40	1.01 [0.88, 1.17]	0.89	0.92	1.01 [0.87, 1.17]	0.94	0.94	ns
IL-12p70	1.38 [1.07, 1.77]	0.01	0.04	1.37 [1.04, 1.80]	0.02	0.05	ns
IL-13	1.25 [1.10, 1.43]	0.001	0.01	1.26 [1.09, 1.45]	0.002	0.01	*
IL-15	2.41 [0.95, 6.12]	0.06	0.12	2.87 [1.15, 7.16]	0.02	0.05	ns
IL-17a	1.16 [0.92, 1.46]	0.20	0.27	1.14 [0.89, 1.45]	0.30	0.37	ns
IL-17f	1.65 [1.22, 2.23]	0.001	0.01	1.76 [1.24, 2.48]	0.001	0.01	*
IL-18	1.45 [1.02, 2.08]	0.04	0.08	1.53 [1.08, 2.17]	0.02	0.04	*
IL-1α	2.18 [1.37, 3.46]	0.001	0.01	2.25 [1.34, 3.80]	0.00	0.01	*
IL-1β	1.09 [0.80, 1.49]	0.59	0.63	1.07 [0.76, 1.49]	0.71	0.75	ns
IL-1Ra	1.24 [0.62, 2.48]	0.54	0.60	1.28 [0.64, 2.53]	0.49	0.56	ns
IL-2	1.41 [1.08, 1.85]	0.01	0.04	1.44 [1.07, 1.94]	0.02	0.04	*
IL-22	1.44 [1.08, 1.91]	0.01	0.04	1.54 [1.11, 2.15]	0.01	0.04	*
IL-25	1.47 [1.16, 1.86]	0.002	0.01	1.49 [1.15, 1.93]	0.002	0.01	*
IL-3	2.85 [0.44, 18.34]	0.27	0.33	3.21 [0.50, 20.66]	0.22	0.28	ns
IL-4	1.14 [1.02, 1.28]	0.02	0.05	1.15 [1.02, 1.31]	0.03	0.05	ns
IL-5	1.04 [1.01, 1.06]	0.01	0.04	1.04 [1.01, 1.07]	0.01	0.04	*
IL-6	1.75 [1.26, 2.43]	0.001	0.01	1.84 [1.31, 2.58]	0.0005	0.01	*
IL-8	1.25 [0.91, 1.73]	0.17	0.25	1.31 [0.92, 1.87]	0.14	0.20	ns
IL-9	4.87 [1.36, 17.42]	0.01	0.04	4.72 [1.28, 17.44]	0.02	0.05	*
IP-10	1.06 [0.93, 1.21]	0.37	0.44	1.07 [0.93, 1.23]	0.37	0.44	ns
MCP-1	1.44 [1.07, 1.93]	0.01	0.04	1.45 [1.07, 1.96]	0.02	0.04	*
MIG	1.01 [0.81, 1.26]	0.94	0.94	1.01 [0.79, 1.31]	0.91	0.94	ns
MIP-1α	1.91 [0.82, 4.45]	0.13	0.20	2.11 [0.92, 4.83]	0.08	0.12	ns
MIP-1β	3.23 [1.36, 7.65]	0.01	0.04	3.83 [1.51, 9.68]	0.005	0.02	*
RANTES	1.49 [0.98, 2.28]	0.06	0.12	1.69 [1.01, 2.84]	0.05	0.08	ns
sCD40L	1.16 [0.93, 1.45]	0.19	0.26	1.18 [0.93, 1.51]	0.18	0.25	ns
TNF-α	1.61 [1.21, 2.15]	0.001	0.01	1.75 [1.24, 2.46]	0.001	0.01	*
VEGF-A	1.02 [0.96, 1.09]	0.50	0.57	1.01 [0.95, 1.08]	0.68	0.75	ns

The single ICI cohort consisted of monotherapy ICI alone and single ICI in combination with chemotherapy or targeted therapy. All hazard ratios are reported as per increase in fold change. Significance was set at adjusted $P < 0.05$. Abbreviations: Adj. = adjusted, CI = confidence interval, ICI = immune checkpoint inhibitor, ns = non-significant.

[A]Hazard ratios were adjusted for age, gender, race, prior oncologic treatment, and disease stage.

Table S7. Characteristics of the early on treatment cytokine cohort: Wilcoxon analysis.

Characteristics	Total patients (*n* = 62)
**Age on study (years)**	
Median (range)	67 (27-83)
**Sex - no. (%)**	
Female	20 (32.3)
Male	42 (67.7)
**Race - no. (%)**	
White	41 (66.1)
Black	18 (29.0)
Other	3 (4.8)
**Autoimmune history - no. (%)**	
Yes	8(12.9)
No	54 (87.1)
**Cancer type - no. (%)**	
Adrenal	1 (1.6)
Biliary Tract	1 (1.6)
Bladder	5 (8.1)
Breast	2 (3.2)
Cervical	0 (0.0)
Colorectal	0 (0.0)
Endometrial	1 (1.6)
Esophagogastric Junction	1 (1.6)
Head and Neck	6 (9.7)
Hepatocellular Carcinoma	16 (25.8)
Lung	2 (3.2)
Melanoma	5 (8.1)
Neuroendocrine	1 (1.6)
Pancreas	0 (0.0)
Renal Cell Carcinoma	17 (27.4)
Sarcoma	2 (3.2)
Squamous Cell Carcinoma of Skin	2 (3.3)
Vulvovaginal	0 (0.0)
**Disease stage - no. (%)**	
Early	7 (11.3)
Advanced/Metastatic	55 (88.7)
**Immune checkpoint inhibitor - no. (%)**	
anti-PD-1 or anti-PD-L1	
Atezolizumab	11 (17.7)
Cemiplimab	2 (3.2)
Nivolumab	8 (12.9)
Pembrolizumab	28 (45.2)
anti-CTLA-4 and anti-PD-1	
Ipilimumab + Nivolumab	13 (21.0)
anti-LAG-3 and anti-PD-1	
Relatlimab + Nivolumab	0 (0.0)
**Treatment regimen - no. (%)**	
ICI Monotherapy	20 (32.3)
ICI Combination Therapy	13 (21.0)
ICI with Targeted Therapy or Chemotherapy	29 (46.8)
**Prior systemic therapy - no. (%)**	
Yes	23 (37.1)
No	39 (62.9)
**Prior ICI therapy - no. (%)**	
Yes	4 (6.5)
No	58 (93.5)
**Max irAE status - no. (%)**	
Grade 2 or higher irAE	34 (54.8)
No grade 2 or higher irAE	28 (45.2)

Table S8. Wilcoxon analysis: early on treatment cytokine and irAE

	Grade 2 or higher irAE (n=34)		No Grade 2 or higher irAE (n=28)		
Cytokines	Mean	Std dev	Mean	Std dev	P-value
IL-5	4.58	12.80	1.18	1.05	0.01
IL-6	1.32	1.09	0.72	0.47	0.01
IL13	1.59	3.04	0.86	0.41	0.34
IL-25	1.51	1.75	0.87	0.50	0.22
IL-17f	1.29	1.06	0.89	0.37	0.08
TNF-α	1.62	1.25	0.98	0.58	0.02

	Dermatologic irAE (n=5)		No Grade 2 or higher irAE (n=28)		
Cytokines	Mean	Std dev	Mean	Std dev	P-value
IL-5	1.60	1.40	1.18	1.05	0.61
IL-6	1.47	0.43	0.72	0.47	0.004
IL13	1.09	0.20	0.86	0.41	0.33
IL-25	1.38	0.89	0.87	0.50	0.25
IL-17f	1.68	0.96	0.89	0.37	0.04
TNF-α	1.39	0.65	0.98	0.58	0.17

	Enterocolitis irAE (n=6)		No Grade 2 or higher irAE (n=28)		
Cytokines	Mean	Std dev	Mean	Std dev	P-value
IL-5	1.55	0.85	1.18	1.05	0.24
IL-6	1.69	1.33	0.72	0.47	0.03
IL13	1.38	0.95	0.86	0.41	0.07
IL-25	1.45	0.74	0.87	0.50	0.05
IL-17f	1.27	0.45	0.89	0.37	0.07
TNF-α	1.66	1.03	0.98	0.58	0.12

	Endocrine irAE (n=7)		No Grade 2 or higher irAE (n=28)		
Cytokines	Mean	Std dev	Mean	Std dev	P-value
IL-5	13.18	27.75	1.18	1.05	0.03
IL-6	2.22	1.33	0.72	0.47	0.0002
IL-13	1.34	1.30	0.86	0.41	0.91
IL-25	1.63	2.70	0.87	0.50	0.66
IL-17f	1.78	2.07	0.89	0.37	0.10
TNF-α	2.14	2.08	0.98	0.58	0.10

	Pneumonitis irAE (n=5)		No Grade 2 or higher irAE (n=28)		
Cytokines	Mean	Std dev	Mean	Std dev	P-value
IL-5	2.52	2.89	1.18	1.05	0.39
IL-6	0.68	0.37	0.72	0.47	0.98
IL-13	1.00	0.00003	0.86	0.41	0.42
IL-25	1.58	1.71	0.87	0.50	0.55
IL-17f	0.80	0.27	0.89	0.37	0.66
TNF-α	1.93	1.25	0.98	0.58	0.03

	Other irAE (n=11)		No Grade 2 or higher irAE (n=26)		
Cytokines	Mean	Std dev	Mean	Std dev	P-value
IL-5	3.04	2.73	1.18	1.05	0.04
IL-6	0.78	0.80	0.72	0.47	0.72
IL-13	2.37	5.30	0.86	0.41	0.73
IL-25	1.51	1.97	0.87	0.50	0.56
IL-17f	1.03	0.42	0.89	0.37	0.74
TNF-α	1.24	0.88	0.98	0.58	0.55

Results of two-tailed Wilcoxon tests (*P*-value) for selected cytokines. Mean and standard deviation are reported for fold change. To address survivorship bias, patients were only designated as having no grade 2 or higher irAE if they had at least 6 months of follow up. Abbreviations: Std dev = standard deviation.

Table S9. Wilcoxon analysis: early on treatment cytokine and response

	Response (n=26)		No response (n=48)		
Cytokines	Mean	Std dev	Mean	Std dev	P-value
IL-5	1.85	1.65	3.39	10.96	0.39
IL-6	0.79	0.63	1.34	1.20	0.048
IL-13	0.92	0.38	1.35	2.57	0.42
IL-25	0.97	0.88	1.28	1.52	0.40
IL-17f	0.96	0.41	1.18	0.91	0.17
TNF-α	1.16	0.81	1.28	0.95	0.33

Results of two-tailed Wilcoxon tests (p-value) for selected cytokines. Mean and standard deviation are reported for fold change. Abbreviations: Std dev = standard deviation.

Table S10. Early on treatment cytokine cox analysis: cancer-specific survival

Cytokines	Unadjusted Hazard ratio [95% CI]	P-value	Adjusted P-value	Adjusted Hazard ratio [95% CI][A]	P-value	Adjusted P-value	Adj. P-value significance
G-CSF	1.18 [0.86, 1.62]	0.30	0.53	1.15 [0.84, 1.59]	0.38	0.61	ns
GM-CSF	1.02 [0.99, 1.05]	0.18	0.38	1.02 [0.99, 1.05]	0.30	0.60	ns
IFN-γ	1.09 [0.73, 1.62]	0.68	0.84	1.01 [0.66, 1.54]	0.95	0.99	ns
IL-10	1.12 [0.93, 1.35]	0.24	0.48	1.15 [0.91, 1.44]	0.24	0.54	ns
IL-12p40	0.97 [0.73, 1.28]	0.81	0.86	0.91 [0.63, 1.31]	0.62	0.81	ns
IL-12p70	1.32 [1.02, 1.70]	0.04	0.17	1.27 [0.95, 1.72]	0.11	0.44	ns
IL-13	1.18 [1.04, 1.35]	0.01	0.08	1.18 [1.02, 1.37]	0.03	0.19	ns
IL-15	4.49 [1.76, 11.46]	0.002	0.03	5.81 [1.96, 17.26]	0.002	0.02	*
IL-17a	1.15 [0.82, 1.63]	0.41	0.63	1.10 [0.73, 1.65]	0.66	0.81	ns
IL-17f	1.38 [0.95, 2.00]	0.09	0.24	1.32 [0.87, 2.02]	0.20	0.54	ns
IL-18	0.99 [0.96, 1.03]	0.73	0.86	0.99 [0.96, 1.03]	0.64	0.81	ns
IL-1α	1.84 [1.11, 3.04]	0.02	0.09	1.77 [0.98, 3.20]	0.06	0.27	ns
IL-1β	1.13 [0.71, 1.81]	0.60	0.83	1.13 [0.68, 1.88]	0.64	0.81	ns
IL-1Ra	1.57 [0.64, 3.85]	0.33	0.53	1.54 [0.61, 3.92]	0.36	0.61	ns
IL-2	1.32 [0.96, 1.81]	0.09	0.24	1.27 [0.89, 1.79]	0.18	0.54	ns
IL-22	1.04 [0.62, 1.75]	0.87	0.87	1.00 [0.58, 1.73]	0.99	0.99	ns
IL-25	1.30 [1.00, 1.69]	0.05	0.19	1.25 [0.93, 1.68]	0.14	0.50	ns
IL-3	1.88 [0.12, 29.08]	0.65	0.83	2.52 [0.13, 48.22]	0.54	0.78	ns
IL-4	1.11 [0.99, 1.26]	0.09	0.24	1.09 [0.95, 1.26]	0.21	0.54	ns
IL-5	1.03 [1.00, 1.06]	0.05	0.19	1.04 [1.00, 1.08]	0.03	0.19	ns
IL-6	1.91 [1.32, 2.77]	0.001	0.02	1.99 [1.31, 3.04]	0.001	0.02	*
IL-8	1.46 [1.07, 1.99]	0.02	0.09	1.50 [1.03, 2.20]	0.04	0.19	ns
IL-9	1.07 [0.66, 1.73]	0.79	0.86	0.97 [0.58, 1.62]	0.91	0.99	ns
IP-10	0.98 [0.82, 1.16]	0.79	0.86	0.97 [0.81, 1.16]	0.73	0.86	ns
MCP-1	1.24 [1.05, 1.46]	0.01	0.08	1.38 [1.07, 1.76]	0.01	0.13	ns
MIG	1.02 [0.82, 1.27]	0.86	0.87	0.99 [0.78, 1.25]	0.92	0.99	ns
MIP-1α	1.96 [0.87, 4.41]	0.11	0.26	1.75 [0.71, 4.28]	0.22	0.54	ns
MIP-1β	1.89 [0.64, 5.59]	0.25	0.48	1.69 [0.56, 5.11]	0.36	0.61	ns
RANTES	0.59 [0.20, 1.72]	0.33	0.53	0.58 [0.19, 1.81]	0.35	0.61	ns
sCD40L	0.85 [0.44, 1.63]	0.63	0.83	0.78 [0.39, 1.56]	0.47	0.72	ns
TNF-α	1.14 [0.72, 1.81]	0.57	0.83	1.00 [0.59, 1.69]	0.99	0.99	ns
VEGF-A	1.04 [0.98, 1.10]	0.16	0.37	1.04 [0.97, 1.10]	0.26	0.56	ns

All hazard ratios are reported as per increase in fold change. Significance was set at adjusted $P < 0.05$. Abbreviations: Adj. = adjusted, CI = confidence interval, ns = non-significant.
[A]Hazard ratios were adjusted for age, gender, race, treatment with dual ICI, prior oncologic treatment, and disease stage.

Table S11. Early on treatment cytokine cox analysis: overall survival

Cytokines	Unadjusted Hazard ratio [95% CI]	P-value	Adjusted P-value	Adjusted Hazard ratio [95% CI][A]	P-value	Adjusted P-value	Adj. P-value significance
G-CSF	1.08 [0.79, 1.46]	0.64	0.82	1.06 [0.79, 1.42]	0.72	0.91	ns
GM-CSF	1.02 [0.99, 1.04]	0.28	0.53	1.01 [0.98, 1.04]	0.45	0.76	ns
IFN-γ	0.97 [0.65, 1.46]	0.90	0.93	0.93 [0.62, 1.39]	0.73	0.91	ns
IL-10	1.07 [0.90, 1.28]	0.45	0.69	1.01 [0.83, 1.24]	0.92	0.96	ns
IL-12p40	0.94 [0.72, 1.24]	0.68	0.82	0.85 [0.60, 1.21]	0.37	0.75	ns
IL-12p70	1.23 [0.95, 1.60]	0.12	0.35	1.18 [0.89, 1.56]	0.24	0.75	ns
IL-13	1.14 [1.01, 1.29]	0.04	0.22	1.14 [0.99, 1.31]	0.07	0.31	ns
IL-15	3.16 [1.31, 7.62]	0.01	0.11	3.60 [1.39, 9.31]	0.01	0.10	ns
IL-17a	1.06 [0.75, 1.50]	0.73	0.84	0.97 [0.62, 1.49]	0.88	0.96	ns
IL-17f	1.37 [0.99, 1.89]	0.06	0.27	1.30 [0.90, 1.87]	0.16	0.57	ns
IL-18	0.99 [0.97, 1.02]	0.69	0.82	0.99 [0.97, 1.02]	0.54	0.87	ns
IL-1α	1.68 [1.04, 2.71]	0.03	0.21	1.56 [0.91, 2.68]	0.10	0.42	ns
IL-1β	1.00 [0.62, 1.62]	1.00	1.00	1.02 [0.62, 1.68]	0.94	0.96	ns
IL-1Ra	2.41 [1.41, 4.12]	0.001	0.02	2.17 [1.21, 3.88]	0.01	0.10	ns
IL-2	1.21 [0.88, 1.67]	0.25	0.52	1.14 [0.82, 1.59]	0.43	0.76	ns
IL-22	1.06 [0.69, 1.63]	0.80	0.88	1.08 [0.68, 1.72]	0.73	0.91	ns
IL-25	1.19 [0.91, 1.55]	0.20	0.45	1.14 [0.87, 1.49]	0.33	0.75	ns
IL-3	1.82 [0.18, 18.37]	0.61	0.82	2.69 [0.20, 35.60]	0.45	0.76	ns
IL-4	1.09 [0.97, 1.23]	0.14	0.37	1.07 [0.93, 1.22]	0.34	0.75	ns
IL-5	1.02 [0.99, 1.05]	0.12	0.35	1.04 [1.00, 1.07]	0.04	0.22	ns
IL-6	1.74 [1.27, 2.38]	0.001	0.02	1.77 [1.26, 2.49]	0.001	0.04	*
IL-8	1.38 [1.04, 1.83]	0.02	0.19	1.46 [1.03, 2.06]	0.03	0.20	ns
IL-9	1.05 [0.69, 1.59]	0.83	0.88	0.93 [0.59, 1.45]	0.74	0.91	ns
IP-10	1.04 [0.95, 1.15]	0.38	0.64	1.03 [0.93, 1.13]	0.62	0.91	ns
MCP-1	1.17 [0.99, 1.38]	0.07	0.28	1.32 [1.04, 1.67]	0.02	0.17	ns
MIG	1.04 [0.88, 1.23]	0.64	0.82	1.00 [0.83, 1.19]	0.96	0.96	ns
MIP-1α	1.71 [0.79, 3.70]	0.17	0.42	1.45 [0.63, 3.32]	0.38	0.75	ns
MIP-1β	2.09 [0.87, 5.03]	0.10	0.35	1.68 [0.68, 4.15]	0.26	0.75	ns
RANTES	0.73 [0.33, 1.64]	0.44	0.69	0.85 [0.37, 1.97]	0.71	0.91	ns
sCD40L	1.08 [0.77, 1.52]	0.66	0.82	1.04 [0.72, 1.48]	0.85	0.96	ns
TNF-α	1.21 [0.84, 1.73]	0.31	0.54	1.04 [0.68, 1.58]	0.87	0.96	ns
VEGF-A	1.03 [0.97, 1.09]	0.28	0.53	1.03 [0.97, 1.09]	0.37	0.75	ns

All hazard ratios are reported as per increase in fold change. Significance was set at adjusted $P < 0.05$. Abbreviations: Adj. = adjusted, CI = confidence interval, ns = non-significant.
[A]Hazard ratios were adjusted for age, gender, race, treatment with dual ICI, prior oncologic treatment, and disease stage.

Table S12. Characteristics of the time of irAE cytokine cohort: Wilcoxon analysis.

Characteristics	Total patients (*n* = 55)
**Age on study (years)**	
Median (range)	65 (27-83)
**Sex - no. (%)**	
Female	17 (30.9)
Male	38 (69.1)
**Race - no. (%)**	
White	34 (61.8)
Black	18 (32.7)
Other	3 (5.5)
**Autoimmune history - no. (%)**	
Yes	8 (14.5)
No	47 (85.5)
**Cancer type - no. (%)**	
Adrenal	0 (0.0)
Biliary Tract	1 (1.8)
Bladder	5 (9.1)
Breast	1 (1.8)
Cervical	0 (0.0)
Colorectal	0 (0.0)
Endometrial	1 (1.8)
Esophagogastric Junction	1 (1.8)
Head and Neck	5 (9.1)
Hepatocellular Carcinoma	17 (30.9)
Lung	2 (3.6)
Melanoma	4 (7.3)
Neuroendocrine	1 (1.8)
Pancreas	0 (0.0)
Renal Cell Carcinoma	16 (29.1)
Sarcoma	1 (1.9)
Squamous Cell Carcinoma of Skin	0 (0.0)
Vulvovaginal	0 (0.0)
**Disease stage - no. (%)**	
Early	6 (10.9)
Advanced/Metastatic	49 (89.1)
**Immune checkpoint inhibitor - no. (%)**	
anti-PD-1 or anti-PD-L1	
Atezolizumab	10 (18.2)
Cemiplimab	0 (0.0)
Nivolumab	7 (12.7)
Pembrolizumab	23 (41.8)
anti-CTLA-4 and anti-PD-1	
Ipilimumab + Nivolumab	14 (25.5)
anti-LAG-3 and anti-PD-1	
Relatlimab + Nivolumab	1 (1.8)
**Treatment regimen - no. (%)**	
ICI Monotherapy	17 (30.9)
ICI Combination Therapy	15 (27.3)
ICI with Targeted Therapy or Chemotherapy	23 (41.8)
**Prior systemic therapy - no. (%)**	
Yes	21 (38.2)
No	34 (61.8)
**Prior ICI therapy - no. (%)**	
Yes	5 (9.1)
No	50 (90.9)
**Max irAE status - no. (%)**	
At least one grade 2 or higher irAE	24 (43.6)
No grade 2 or higher irAE	31 (56.4)

Table S13. Wilcoxon analysis: time of irAE cytokines

	Grade 2 or higher irAE (n=24)		No Grade 2 or higher irAE (n=31)		
Cytokines	Mean	Std dev	Mean	Std dev	P-value
IL-5	4.92	15.20	1.18	1.00	0.07
IL-6	2.89	3.08	0.68	0.46	0.00001
IL-13	1.30	1.17	0.88	0.40	0.14
IL-25	2.54	7.15	0.86	0.48	0.43
IL-17f	1.23	0.73	0.89	0.36	0.01
TNF-α	2.16	2.50	1.00	0.58	0.06

	Dermatologic irAE (n=6)		No Grade 2 or higher irAE (n=31)		
Cytokines	Mean	Std dev	Mean	Std dev	P-value
IL-5	1.86	1.16	1.18	1.00	0.19
IL-6	1.88	0.87	0.68	0.46	0.0003
IL-13	1.23	0.47	0.88	0.40	0.14
IL-25	0.82	0.25	0.86	0.48	0.80
IL-17f	1.30	0.57	0.89	0.36	0.04
TNF-α	1.50	0.62	1.00	0.58	0.07

	Enterocolitis irAE (n=3)		No Grade 2 or higher irAE (n=31)		
Cytokines	Mean	Std dev	Mean	Std dev	P-value
IL-5	0.96	0.34	1.18	1.00	1.00
IL-6	5.75	5.92	0.68	0.46	0.01
IL-13	1.19	0.33	0.88	0.40	0.29
IL-25	1.56	0.15	0.86	0.48	0.02
IL-17f	2.09	1.75	0.89	0.36	0.04
TNF-α	2.68	2.93	1.00	0.58	0.41

	Endocrine irAE (n=5)		No Grade 2 or higher irAE (n=31)		
Cytokines	Mean	Std dev	Mean	Std dev	P-value
IL-5	16.49	33.19	1.18	1.00	0.07
IL-6	1.79	1.29	0.68	0.46	0.03
IL-13	0.73	0.38	0.88	0.40	0.35
IL-25	0.67	0.19	0.86	0.48	0.27
IL-17f	1.06	0.08	0.89	0.36	0.07
TNF-α	1.89	1.80	1.00	0.58	0.32

	Pneumonitis irAE (n=4)		No Grade 2 or higher irAE (n=31)		
Cytokines	Mean	Std dev	Mean	Std dev	P-value
IL-5	0.86	0.78	1.18	1.00	0.56
IL-6	2.30	1.98	0.68	0.46	0.07
IL-13	1.31	0.62	0.88	0.40	0.13
IL-25	9.51	17.62	0.86	0.48	0.78
IL-17f	0.89	0.22	0.89	0.36	0.95
TNF-α	3.13	4.99	1.00	0.58	0.94

	Other irAE (n=6)		No Grade 2 or higher irAE (n=26)		
Cytokines	Mean	Std dev	Mean	Std dev	P-value
IL-5	3.00	2.70	1.18	1.00	0.07
IL-6	3.77	4.09	0.68	0.46	0.06
IL-13	1.90	2.22	0.88	0.40	0.34
IL-25	1.68	1.30	0.86	0.48	0.17
IL-17f	1.11	0.54	0.89	0.36	0.13
TNF-α	2.12	2.42	1.00	0.58	0.19

Results of two-tailed Wilcoxon tests (p-value) for selected cytokines. Mean and standard deviation are reported for fold change. To address survivorship bias, patients were only designated as having no grade 2 or higher irAE if they had at least 6 months of follow up. Abbreviations: Std dev = standard deviation.

Table S14. CyTOF antibody panel

The CyTOF antibody marker panel is displayed in the Supplemental 14 excel file.

Table S15. CyTOF clustering annotations

The CyTOF clustering annotation table is displayed in the Supplemental 15 excel file.

Table S16. Characteristics of the overall CyTOF cohort.

Characteristics	Total patients (*n* = 99)
**Age on study (years)**	
Median (range)	65 (20-87)
**Sex - no. (%)**	
Female	36 (36.4)
Male	63 (63.6)
**Race - no. (%)**	
White	67 (67.7)
Black	25 (25.3)
Other	7 (7.1)
**Autoimmune history - no. (%)**	
Yes	13 (13.1)
No	86 (86.9)
**Cancer type - no. (%)**	
Adrenal	1 (1.0)
Biliary Tract	2 (2.0)
Bladder	10 (10.1)
Breast	3 (3.0)
Cervical	2 (2.0)
Colorectal	1 (1.0)
Endometrial	1 (1.0)
Esophagogastric Junction	1 (1.0)
Head and Neck	7 (7.1)
Hepatocellular Carcinoma	26 (26.3)
Lung	2 (2.0)
Melanoma	9 (9.1)
Neuroendocrine	2 (2.0)
Pancreas	0 (0)
Renal Cell Carcinoma	24 (24.3)
Sarcoma	3 (3.0)
Squamous Cell Carcinoma of Skin	4 (4.1)
Vulvovaginal	1 (1.0)
**Disease stage - no. (%)**	
Early	11 (11.1)
Advanced/Metastatic	88 (88.9)
**Immune checkpoint inhibitor - no. (%)**	
anti-PD-1 or anti-PD-L1	
Atezolizumab	16 (16.2)
Cemiplimab	4 (4.0)
Nivolumab	14 (14.2)
Pembrolizumab	42 (42.4)
anti-CTLA-4 and anti-PD-1	
Ipilimumab + Nivolumab	21 (21.2)
anti-LAG-3 and anti-PD-1	
Relatlimab + Nivolumab	2 (2.0)
**Treatment regimen - no. (%)**	
ICI Monotherapy	40 (40.4)
ICI Combination Therapy	23 (23.2)
ICI with Targeted Therapy or Chemotherapy	36 (36.4)
**Prior systemic therapy - no. (%)**	
Yes	37 (37.4)
No	62 (62.6)
**Prior ICI therapy - no. (%)**	
Yes	3 (3.0)
No	96 (97.0)
**Max irAE status - no. (%)**	
Grade 2 or higher irAE	43 (43.4)
No grade 2 or higher irAE	56 (56.6)

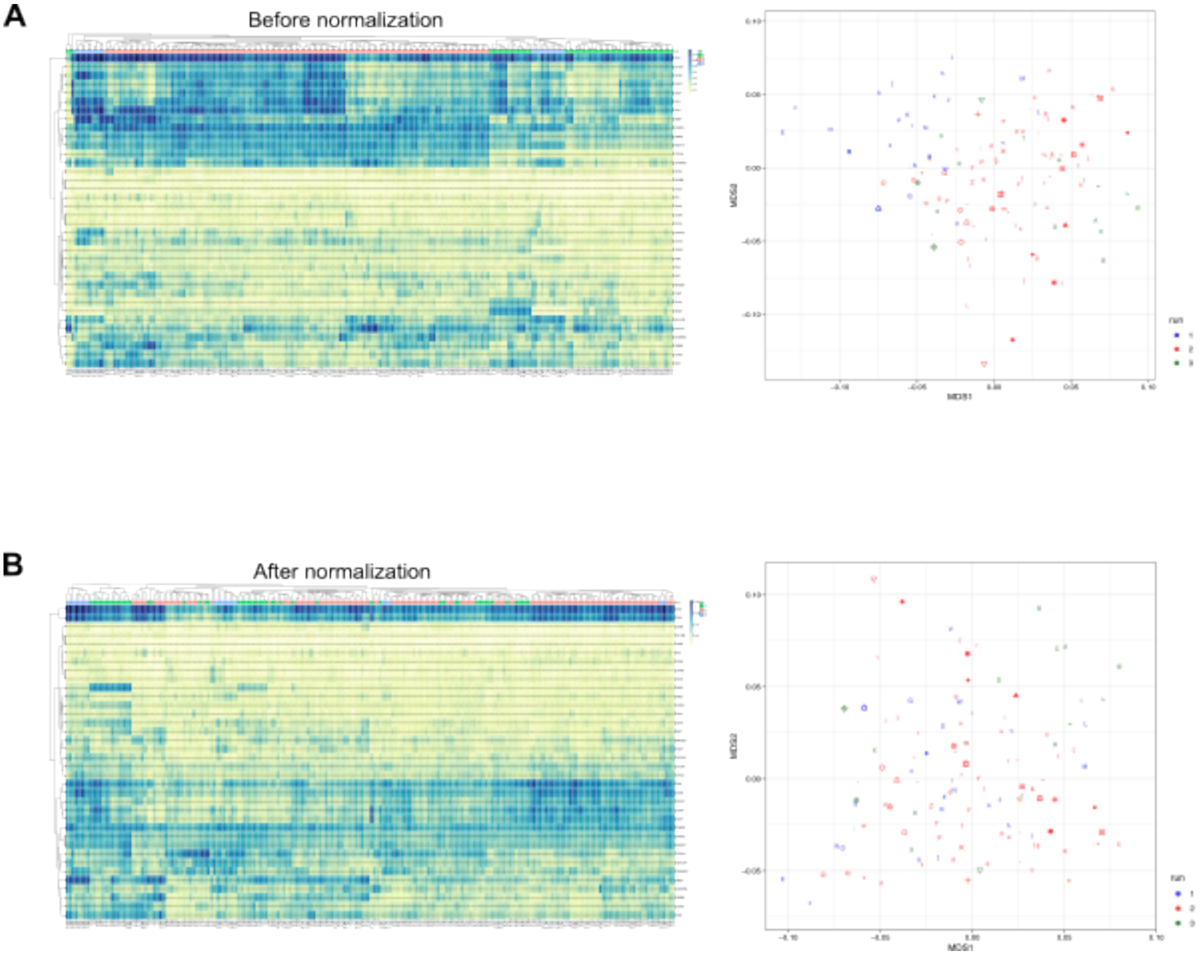

Figure S4. Diagnostic plots indicating success of CyTOF sample normalization. Heatmap indicating mean expression of panel markers on a per sample basis. Heatmap is clustered by sample ID (columns). Alongside the heatmap is a multi-dimensional scaling (MDS) plot indicating similarity of samples colored by run. Plots are **(A)** before and **(B)** after normalization by CytoNorm.

Table S17. CyTOF analysis: baseline

	Grade 2 or higher irAE		No grade 2 or higher irAE			
**Cluster**	**Mean**	**Std dev**	**Mean**	**Std dev**	**P-value**	**Adj. P-value**
TcEFF_I	1.094481161	2.105745367	0.550008231	0.571846671	0.067	0.437
TcEFF_II	8.069102211	7.462278938	8.224636036	8.323618685	0.92	0.990
TcEFF_III	1.260526371	1.429231469	0.983253442	1.025288948	0.26	0.751
TcEM	1.672661981	1.426621488	1.674867615	1.399174549	0.99	0.990
TcCM	2.246572411	1.875856033	2.366168939	2.792520948	0.81	0.927
TcN	3.34480257	3.572421133	4.28481827	3.306516169	0.18	0.669
ThCTL	2.341773666	3.163386868	2.534514689	4.958162959	0.82	0.927
Th1	0.051126376	0.224945699	0.033856701	0.193533732	0.68	0.923
Th1EM	2.968580862	2.24244553	2.256665801	1.45089405	0.059	0.437
Th2	0.701334381	0.968119629	0.502781078	0.710710746	0.24	0.751
Th2EM	2.413044394	1.208046626	2.183187587	1.288382889	0.37	0.802
Th2CM_I	0.329795046	0.701845023	0.269288511	0.873422701	0.71	0.923
Th2CM_II	10.69760296	4.628626052	11.09732158	5.597577887	0.71	0.923
Th2CM_III	2.901053621	1.886137192	2.241096679	1.717665967	0.072	0.437
Th17	1.011418008	0.947144627	0.69569835	0.459017956	0.031	0.437
Treg	1.330516125	0.545719769	1.325055839	0.649659531	0.96	0.990
ThN	13.3928927	9.791113693	14.15356527	9.797296405	0.7	0.923
DNT_I	0.209658734	0.169766212	0.170275755	0.109906603	0.17	0.669
DNT_II	0.203419944	1.069222697	0.054442613	0.145144966	0.31	0.756
DNT_III	1.117637788	2.064862001	0.789824255	1.13924966	0.32	0.756
DNT_IV	1.134191724	2.150430466	0.600883848	0.680656433	0.084	0.437
NK_I	0.599052363	0.686053217	0.528605356	0.504201802	0.56	0.923
NK_II	9.719629499	7.001327611	10.38750201	6.893315457	0.64	0.923
NK_III	0.621977386	0.485968183	0.646542254	0.528119395	0.81	0.927
B	6.868561535	5.776636688	6.430472359	4.058156415	0.66	0.923
Myeloid	23.38540451	15.34972298	24.71208774	13.12368959	0.64	0.923

Results of two sample t-tests (p-value) for all immune cell clusters. Mean and standard deviation are reported as percentage of acquired cells. Abbreviations: Adj. = adjusted, CTL = cytotoxic T lymphocyte, CM = central memory, DNT = double-negative T, EFF = effector, EM = effector memory, N = naïve, NK = natural killer, PBMC = peripheral blood mononuclear cell, Std dev = standard deviation, Tc = cytotoxic T cell, Th = helper T cell.

Table S18. CyTOF analysis: on treatment

	Grade 2 or higher irAE		No grade 2 or higher irAE			
Cluster	Mean	Std dev	Mean	Std dev	P-value	Adj. P-value
TcEFF_I	0.824986872	0.904987344	0.683002689	1.116783115	0.50	0.894
TcEFF_II	9.760018777	8.698985093	9.065992725	9.496050375	0.71	0.900
TcEFF_III	1.372687798	1.53062144	1.230960848	1.414044954	0.63	0.900
TcEM	1.474537364	1.4002898	1.407265702	1.217507546	0.80	0.900
TcCM	2.048377809	1.873684684	2.10802384	2.458545828	0.9	0.900
TcN	2.95429758	3.526131608	3.887899564	3.250004622	0.18	0.585
ThCTL	2.550959934	3.330741886	2.68307217	5.160283817	0.88	0.900
Th1	0.060374037	0.255731522	0.03281159	0.18203804	0.53	0.894
Th1EM	2.534288866	1.972636343	1.89943556	1.303901431	0.057	0.436
Th2	0.512492033	0.612683438	0.447735694	0.646133322	0.61	0.900
Th2EM	2.854287273	2.100762878	2.031459656	1.053609548	0.012	0.156
Th2CM_I	0.307199381	0.687727099	0.28059493	0.893284204	0.87	0.900
Th2CM_II	10.35797965	5.501101671	10.1132219	5.126857965	0.82	0.900
Th2CM_III	2.773441226	2.558488451	2.390855604	1.857174881	0.39	0.845
Th17	1.500296974	1.457452779	0.741718065	0.434322877	0.00037	0.00962
Treg	1.86914049	0.962667818	1.55245386	0.740631315	0.067	0.436
ThN	11.84758338	9.448471865	13.06491212	9.419336801	0.53	0.894
DNT_I	0.297346694	0.264497416	0.234155479	0.146791382	0.13	0.483
DNT_II	0.127034725	0.621737293	0.041994782	0.094546111	0.32	0.832
DNT_III	1.268777914	3.716902778	0.691899181	0.908899781	0.27	0.780
DNT_IV	1.249410076	2.203046525	0.73951559	1.018643196	0.13	0.483
NK_I	0.777587003	0.961691621	0.72481768	0.604205909	0.74	0.900
NK_II	10.5856385	7.081190484	11.9535633	7.545917463	0.36	0.845
NK_III	0.444069717	0.399254889	0.622415141	0.577755173	0.087	0.452
B	5.692079321	3.966711454	6.274242623	5.325862188	0.55	0.894
Myeloid	23.66570062	15.18354575	24.80948659	14.13616945	0.70	0.900

Results of two sample t-tests (p-value) for all immune cell clusters. Mean and standard deviation are reported as percentage of acquired cells. Abbreviations: Adj. = adjusted, CTL = cytotoxic T lymphocyte, CM = central memory, DNT = double-negative T, EFF = effector, EM = effector memory, N = naïve, NK = natural killer, PBMC = peripheral blood mononuclear cell, Std dev = standard deviation, Tc = cytotoxic T cell, Th = helper T cell.

Table S19. CyTOF analysis: fold change

	Grade 2 or higher irAE		No grade 2 or higher irAE			
Cluster	Mean	Std dev	Mean	Std dev	P-value	Adj. P-value
TcEFF_I	2.138681112	2.793707963	2.382286692	6.286518855	0.81	0.988
TcEFF_II	2.096175371	5.364347227	1.212028623	0.564571099	0.22	0.715
TcEFF_III	1.995453622	3.820060863	1.475736019	1.108125559	0.34	0.884
TcEM	0.947036583	0.4937256	0.958093694	0.67942863	0.93	0.988
TcCM	0.986672417	0.409080824	0.994582055	0.388295715	0.92	0.988
TcN	0.891288323	0.381156594	0.895515809	0.278499881	0.95	0.988
ThCTL	1.767550239	2.373543089	1.240731528	0.788838643	0.13	0.483
Th1	0.858845839	0.888213595	1.486943775	3.022554538	0.30	0.867
Th1EM	0.899737211	0.492776788	0.865308915	0.362508038	0.69	0.988
Th2	1.48492168	3.14202994	1.163940596	0.856435409	0.47	0.929
Th2EM	1.308604896	0.914978505	1.011753919	0.359927193	0.029	0.377
Th2CM_I	0.931691406	0.607549415	1.22933177	1.112546167	0.12	0.483
Th2CM_II	0.990268794	0.341776965	0.944088438	0.300356641	0.48	0.929
Th2CM_III	1.083592655	1.132326528	1.127449974	0.566505042	0.80	0.988
Th17	1.901457602	1.842944811	1.265258571	0.814029622	0.023	0.377
Treg	1.619809501	1.120059442	1.289522693	0.658619237	0.07	0.455
ThN	0.87904462	0.322128539	0.921735083	0.307615571	0.50	0.929
DNT_I	1.616898401	0.85052643	1.583695157	1.135685011	0.87	0.988
DNT_II	1.770141529	4.163757526	3.886385207	14.55227353	0.41	0.929
DNT_III	1.060689919	0.671434348	1.006085149	0.482482068	0.64	0.988
DNT_IV	1.538970679	1.798295953	1.760179508	2.949194488	0.67	0.988
NK_I	1.729647237	1.828295354	1.707519978	1.447509531	0.95	0.988
NK_II	1.265296121	0.907874154	1.245679027	0.534440788	0.89	0.988
NK_III	0.850683646	0.537812181	1.230394381	1.158206106	0.049	0.425
B	1.037522132	0.794137947	1.038074846	0.585825249	1	1
Myeloid	2.032230607	4.46156534	1.125737588	0.539866398	0.13	0.483

Results of two sample t-tests (p-value) for all immune cell clusters. Mean and standard deviation are reported as fold change in percentage of acquired cells. Abbreviations: Adj. = adjusted, CTL = cytotoxic T lymphocyte, CM = central memory, DNT = double-negative T, EFF = effector, EM = effector memory, N = naïve, NK = natural killer, PBMC = peripheral blood mononuclear cell, Std dev = standard deviation, Tc = cytotoxic T cell, Th = helper T cell.

References

94. Charmsaz S, et al. A global live cell barcoding approach for multiplexed mass cytometry profiling of mouse tumors. *JCI Insight*. 2021;6(7).

95. Van Gassen S, et al. CytoNorm: A Normalization Algorithm for Cytometry Data. *Cytometry A*. 2020;97(3):268-278.

96. Van Gassen S, et al. FlowSOM: Using self-organizing maps for visualization and interpretation of cytometry data. *Cytometry A*. 2015;87(7):636-645.

Key Resources

Software and R packages	Source	Identifier
Cairo R package	Urbanek S, Horner J (2022). _Cairo: R Graphics Device using Cairo Graphics Library for Creating High-Quality Bitmap (PNG, JPEG, TIFF), Vector (PDF, SVG, PostScript) and Display (X11 and Win32) Output_.	https://CRAN.R-project.org/package=Cairo
circlize R package	Gu, Z. (2014) circlize implements and enhances circular visualization in R. Bioinformatics.	https://cran.r-project.org/web/packages/circlize/index.html
ComplexHeatmap R package	Gu, Z. (2016) Complex heatmaps reveal patterns and correlations in multidimensional genomic data. Bioinformatics.	https://CRAN.R-project.org/package=ComplexHeatmap
ConsensusClusterPlus R package	Wilkerson, D. M, Hayes, Neil D (2010). “ConsensusClusterPlus: a class discovery tool with confidence assessments and item tracking.” *Bioinformatics*, **26**(12), 1572-1573.	https://bioconductor.org/packages/release/bioc/html/ConsensusClusterPlus.html
corrplot R package	Taiyun Wei and Viliam Simko (2021). R package 'corrplot': Visualization of a Correlation Matrix (Version 0.92).	https://cran.r-project.org/web/packages/corrplot/index.html
CytoNorm	Van Gassen S, Gaudilliere B, Angst MS, Saeys Y, Aghaeepour N. CytoNorm: A Normalization Algorithm for Cytometry Data. *Cytom Part J Int Soc Anal Cytol*. 2020;97(3):268-278. doi:10.1002/cyto.a.23904	https://github.com/saeyslab/CytoNorm

dplyr R package	Wickham H, François R, Henry L, Müller K, Vaughan D (2023). _dplyr: A Grammar of Data Manipulation_.	https://CRAN.R-project.org/package=dplyr
flowCore R package	Ellis B, Haaland P, Hahne F, Le Meur N, Gopalakrishnan N, Spidlen J, Jiang M, Finak G (2023). *flowCore: flowCore: Basic structures for flow cytometry data*.	https://bioconductor.org/packages/release/bioc/html/flowCore.html
FlowSOM R package	Van Gassen S, Callebaut B, Van Helden M, Lambrecht B, Demeester P, Dhaene T, Saeys Y (2015). "FlowSOM: Using self-organizing maps for visualization and interpretation of cytometry data." *Cytometry Part A*, **87**(7), 636-645.	https://bioconductor.org/packages/release/bioc/html/FlowSOM.html
forestplot R package	Gordon M, Lumley T (2022). _forestplot: Advanced Forest Plot Using 'grid' Graphics.	https://CRAN.R-project.org/package=forestplot
ggplot2 R package	H. Wickham. ggplot2: Elegant Graphics for Data Analysis. Springer-Verlag New York, 2016.	https://ggplot2.tidyverse.org
ggpubr R package	Kassambara A (2023). _ggpubr: 'ggplot2' Based Publication Ready Plots_.	https://cran.r-project.org/web/packages/ggpubr/index.html
ggrepel R package	Slowikowski K (2023). _ggrepel: Automatically Position Non-Overlapping Text Labels with 'ggplot2'_.	https://cran.r-project.org/web/packages/ggrepel/index.html
ggridges R package	Wilke C (2022). _ggridges: Ridgeline Plots in 'ggplot2'_.	https://cran.r-project.org/web/packages/ggridges/index.html
ggsci R package	Xiao N (2023). _ggsci: Scientific Journal and Sci-Fi Themed Color Palettes for 'ggplot2'_.	https://CRAN.R-project.org/package=ggsci
greekLetters package	Rodrigues, K. A. S. (2023). greekLetters: routines for writing Greek letters and	https://CRAN.R-project.org/package=greekLetters

	mathematical symbols on the RStudio and RGui (version 1.0.2).	
here R package	Müller K (2020). _here: A Simpler Way to Find Your Files_.	https://CRAN.R-project.org/package=here
Hmisc R package	Harrell Jr F (2023). _Hmisc: Harrell Miscellaneous_.	https://cran.r-project.org/web/packages/Hmisc/index.html
limma R package	Ritchie ME, Phipson B, Wu D, Hu Y, Law CW, Shi W, Smyth GK (2015). "limma powers differential expression analyses for RNA-sequencing and microarray studies." *Nucleic Acids Research*, **43**(7), e47.	https://bioconductor.org/packages/release/bioc/html/limma.html
matrixStats R package	Bengtsson H (2023). _matrixStats: Functions that Apply to Rows and Columns of Matrices (and to Vectors)_.	https://cran.rstudio.com/web/packages/matrixStats/index.html
openxlsx R package	Schauberger P, Walker A (2023). _openxlsx: Read, Write and Edit xlsx Files_.	https://CRAN.R-project.org/package=openxlsx
pals R package	Wright K (2023). _pals: Color Palettes, Colormaps, and Tools to Evaluate Them_.	https://cran.r-project.org/web/packages/pals/index.html
pheatmap R package	Kolde R (2019). _pheatmap: Pretty Heatmaps_.	https://cran.r-project.org/web/packages/pheatmap/index.html
Prism v10	GraphPad	https://www.graphpad.com/
randomcoloR R package	Ammar R (2019). _randomcoloR: Generate Attractive Random Colors_.	https://cran.r-project.org/web/packages/randomcoloR/index.html
raster R package	Hijmans R (2023). _raster: Geographic Data Analysis and Modeling_.	https://cran.r-project.org/web/packages/raster/index.html
RColorBrewer R package	Neuwirth E (2022). _RColorBrewer: ColorBrewer Palettes_.	https://cran.r-project.org/web/packages/RColorBrewer/index.html
readxl R package	Wickham H, Bryan J (2023). _readxl: Read Excel Files_.	https://cran.r-project.org/web/packages/readxl/index.html
reshape2 R package	Hadley Wickham (2007). Reshaping Data with the reshape	https://cran.r-project.org/web/packages/reshape2/index.html

	Package. Journal of Statistical Software, 21(12), 1-20.	
scales R package	Wickham H, Seidel D (2022). _scales: Scale Functions for Visualization_.	https://cran.r-project.org/web/packages/scales/index.html
stringr R package	Wickham H (2022). _stringr: Simple, Consistent Wrappers for Common String Operations_.	https://CRAN.R-project.org/package=stringr
survival R package	Therneau T (2023). _A Package for Survival Analysis in R_.	https://CRAN.R-project.org/package=survival
survminer R package	Kassambara A, Kosinski M, Biecek P (2021). _survminer: Drawing Survival Curves using 'ggplot2'_.	https://CRAN.R-project.org/package=survminer
tidyverse R package	Wickham H, et. al (2019). "Welcome to the tidyverse." _Journal of Open Source Software_, *4*(43), 1686. doi:10.21105/joss.01686	https://doi.org/10.21105/joss.01686
umap R package	Konopka T (2023). _umap: Uniform Manifold Approximation and Projection_.	https://cran.r-project.org/web/packages/umap/index.html
viridis R package	Simon Garnier, Noam Ross, Robert Rudis, Antônio P. Camargo, Marco Sciaini, and Cédric Scherer (2023). viridis(Lite) - Colorblind-Friendly Color Maps for R. viridis package version 0.6.4.	https://CRAN.R-project.org/package=viridis
